# Brazilian Propolis in the Context of Pathogens Associated with Neonatal Infections: An Antibacterial and Antiviral Approach

**DOI:** 10.3390/pathogens15090922

**Published:** 2026-09-01

**Authors:** Anna Livia Oliveira Santos, Nagela Bernadelli Sousa Silva, Júlia Gomes Teixeira, Gabriel Guimarães Calefi, Júlia Amaral Vieira D’Almeida, Natasha Marques Cassani, Ana Carolina Gomes Jardim, Ralciane de Paula Menezes, Marcelo José Barbosa Silva, Matheus Hikaru Tanimoto, Victor Pena Ribeiro, Jairo Kenupp Bastos, Carlos Henrique Gomes Martins

**Affiliations:** 1 Laboratory of Antimicrobial Testing (LEA), Institute of Biomedical Sciences (ICBM), Universidade Federal de Uberlândia (UFU), Uberlândia 38405-315, Minas Gerais, Brazil; anna.livia@ufu.br (A.L.O.S.); nagela.silva@ufu.br (N.B.S.S.); julia.teixeira1@ufu.br (J.G.T.); ggcalefi@ufu.br (G.G.C.); 2 Antiviral Research Laboratory (LAPAV), Institute of Biomedical Sciences (ICBM), Universidade Federal de Uberlândia (UFU), Uberlândia 38400-902, Minas Gerais, Brazil; juu_amaral@hotmail.com (J.A.V.D.); natashacassani@ufu.br (N.M.C.); jardim@ufu.br (A.C.G.J.); 3 Institute of Biomedical Sciences (ICBM), Universidade Federal de Uberlândia (UFU), Uberlândia 38405-320, Minas Gerais, Brazil; ralciane@ufu.br (R.d.P.M.); majbsilva@ufu.br (M.J.B.S.); 4 Faculty of Pharmaceutical Sciences of Ribeirão Preto, University of São Paulo (USP), Ribeirão Preto 14040-903, São Paulo, Brazil; tanimoto@usp.br (M.H.T.); victor.ribeiro@usda.gov (V.P.R.); jkbastos@fcfrp.usp.br (J.K.B.)

**Keywords:** antibiofilm activity, antimicrobial activity, anti-Zika virus activity, Brazilian red propolis, Caatinga green propolis, *Caenorhabditis elegans*, neonatal infections

## Abstract

Neonatal infections remain a major global health challenge because of antimicrobial resistance, biofilm-associated persistence, and limited antiviral therapies. This study evaluated the antimicrobial, antibiofilm, in vivo efficacy, and antiviral activities of Brazilian red propolis (BRP) and Brazilian green propolis from the “Caatinga” biome (BGP-C) against pathogens associated with neonatal infections. Antimicrobial activity was determined by broth microdilution, whereas antibiofilm activity was assessed through biofilm biomass and metabolic activity inhibition and structural alterations were evaluated by fluorescence microscopy, and scanning electron microscopy. In vivo efficacy was assessed in a *Caenorhabditis elegans* infection model, and antiviral activity was investigated against Zika virus (ZIKV, PE243 strain). Both extracts exhibited antibacterial activity, mainly against Gram-positive bacteria, with minimum inhibitory concentrations of 6.25–400 µg/mL and minimum bactericidal concentrations of 25 to >400 µg/mL. MBIC_50_ and IC_50_ values ranged from 1.56–400 µg/mL and 0.64–163 µg/mL, respectively. Microscopy findings suggested reduced cell viability and compromised membrane integrity, together with biofilm disorganization. BRP consistently increased nematode survival across the infection models in which it was tested, whereas BGP-C showed no consistent protective effect. Under non-cytotoxic conditions, BRP and BGP-C reduced ZIKV infectivity by approximately 34% and 40%, respectively. These findings support the antimicrobial and antiviral potential of both extracts, while BRP demonstrated more consistent in vivo efficacy.

## 1. Introduction

Neonatal infections are those that affect newborns during the first 28 days of life, a period recognized as the most vulnerable according to the World Health Organization [1]. According to the time of onset, these infections are mainly classified as early-onset or late-onset infections [2,3].

At the global level, infections remain among the leading causes of mortality in the neonatal period and are associated with approximately 570,000 deaths due to severe complications such as sepsis, meningitis, neurodevelopmental impairment, and stillbirth. They continue to account for a substantial proportion of deaths in children under five years of age, particularly in low- and middle-income countries, despite the overall global trend toward reduced childhood mortality [4,5,6,7].

These infections can be caused by bacterial, viral, and fungal pathogens. However, bacteria remain the main microorganisms associated with severe outcomes in the neonatal period, particularly in early-onset sepsis (EOS) and late-onset sepsis (LOS) syndromes [8,9,10]. Among the most relevant bacterial agents are *Streptococcus agalactiae* (GBS) and *Listeria monocytogenes*, which are frequently associated with early-onset infections, as well as coagulase-negative staphylococci, such as *Staphylococcus epidermidis*. Gram-negative bacteria, including *Escherichia coli*, *Klebsiella pneumoniae*, *Enterobacter* spp., and *Pseudomonas aeruginosa*, are also important causes of neonatal infections and are associated with high mortality rates and increasing antimicrobial resistance [11,12]. Although less frequent, *S. pneumoniae* can also cause severe neonatal infections. Late-onset infections are frequently healthcare-associated infections and are particularly relevant in neonatal intensive care units (NICUs), where they significantly contribute to neonatal morbidity and mortality and are often associated with the use of invasive medical devices [13,14,15]. Fungal pathogens, particularly *Candida* spp., are also important causes of late-onset infections, especially among premature newborns and those requiring prolonged hospitalization and invasive procedures [16].

In addition to the intrinsic virulence of these pathogens, their ability to adhere to surfaces and form biofilms represents a key determinant of microbial persistence in hospital environments. In NICUs, biofilm formation on catheters, probes, and other invasive devices is directly associated with persistent infections, therapeutic failure, and late-onset sepsis [17,18,19].

Beyond bacterial infections, which represent the main clinical challenge in the neonatal period, viral agents such as Zika virus (ZIKV), an arbovirus of the genus *Orthoflavivirus*, also play a relevant role in this context. Maternal ZIKV infection during pregnancy is associated with severe congenital abnormalities, including neurological, visual, and auditory impairments [20]. The absence of licensed specific antivirals for the treatment of Zika fever reinforces the need for studies aimed at identifying new compounds with antiviral activity, including natural products as a complementary approach [21,22].

Given the challenges posed by bacterial resistance, biofilm formation, and the lack of specific antiviral therapies, the need for new therapeutic alternatives against these pathogens becomes evident. In this scenario, Brazilian propolis stands out as a promising source of bioactive compounds with antibacterial, antibiofilm, and antiviral potential [23,24].

Brazilian red propolis (BRP) is a resinous material produced by *Apis mellifera* bees from plant exudates collected mainly from *Dalbergia ecastaphyllum* and *Symphonia globulifera*. Its botanical origin gives BRP a characteristic chemical profile rich in lipophilic phenolic compounds, particularly isoflavonoids associated with *D. ecastaphyllum* and polyprenylated benzophenones derived from *S. globulifera*, together with prenylated flavonoids, chalcones, and pterocarpans [24,25,26]. Vestitol, medicarpin, and neovestitol are among its major quantified constituents, while liquiritigenin, calycosin, isoliquiritigenin, formononetin, biochanin A, and guttiferone E/xanthochymol have also been identified as relevant chemical markers [25]. These compounds have been associated with the antibacterial activity reported for BRP [24,27,28].

Brazilian green propolis from the “Caatinga” biome (BGP-C), in turn, is predominantly derived from *Mimosa tenuiflora*, commonly known as “jurema-preta,” a plant adapted to the semiarid conditions of northeastern Brazil [29]. Reflecting its distinct botanical source and the characteristic vegetation of the “Caatinga” biome, BGP-C exhibits a phytochemical profile that differs markedly from that of BRP. Its composition is predominantly characterized by flavonoids, particularly flavonols, flavanones, and flavones, as well as chalcones and phenolic acid derivatives [30]. Kaempferide, santin, viscosine, axillarin, tamarixetin, ermanin, kumatakenin, and eriodictyol 5-*O*-methyl ether have been reported among its major quantified flavonoids [31]. Its phytochemical constituents have been associated with the reported anti-HIV [32], anti-inflammatory [33], and antibacterial activities of BGP-C [30,33].

In the context of neonatal infections, studies involving Brazilian propolis remain limited, with a particular scarcity of investigations evaluating BRP and BGP-C against pathogens of neonatal concern. Moreover, experimental evidence regarding their antiviral activity against Zika virus (ZIKV) remains scarce or unavailable. BRP and BGP-C were selected because their distinct botanical origins and phytochemical compositions represent different sources of bioactive compounds. Investigating their respective biological properties therefore broadens the current knowledge of the anti-infective potential of Brazilian propolis and may reveal activities associated with their chemical profiles. Considering these gaps, the present study aimed to evaluate the antimicrobial, antibiofilm, and antiviral activities of BRP and BGP-C, as well as their toxicity and protective efficacy in an in vivo infection model.

## 2. Materials and Methods

### 2.1. The Propolis Extract Acquisition

The Brazilian red propolis (BRP) extract was prepared as described by Santiago et al., (2022) [28] using 200 g of red propolis acquired from the “Cooperativa dos Apicultores de Canavieiras” (COAPER), Canavieiras, Bahia state, Brazil. The sample was subjected to dynamic maceration in a 70% hydroalcoholic ethanol solution at 30 °C and 120 rpm in a shaking incubator (New Brunswick Scientific/Eppendorf, Edison, NJ, USA). After extraction, the material was filtered and the extract was concentrated under vacuum using a rotary evaporator (Büchi, Flawil, St. Gallen, Switzerland), followed by lyophilization until complete dryness, yielding the crude ethanolic BRP extract. The extraction procedure was based on the method previously described [28], and the chemical profile of the extract used in the present study was determined by HPLC-DAD as described below.

The Brazilian green propolis from the “Caatinga” biome (BGP-C) extract was obtained from samples collected in the municipality of Remanso, Bahia, Brazil, produced by Africanized honeybees (*Apis mellifera* L.). The extract was prepared according to the methodology described by Aldana-Mejia et al., (2024) [30]. Approximately 500 g of crude propolis were frozen, ground, and subjected to maceration in a methanol–water solution for 24 h at 30 °C and 120 rpm in a shaking incubator, a procedure performed in two successive extractions. The extracts were combined, concentrated under vacuum using a rotary evaporator, and lyophilized, yielding 180 g of crude extract. The extraction procedure was based on the method previously described, and the chemical profile of the extract used in the present study was determined by HPLC-DAD as described below.

#### HPLC-DAD Analysis of “Caatinga” Green and Red Propolis

The chemical characterization of BGP-C- and BRP extracts used in the present study was performed by high-performance liquid chromatography with diode array detection (HPLC-DAD), using previously developed and validated analytical methods [27,31].

The analysis of BGP-C was performed on a Shimadzu LC-20 Prominence HPLC system equipped with a SIL-10AF autosampler, CTO-20A column oven, DGU-20A3R in-line degasser, CBM-20A communication module, and an SPD-M20A photodiode array detector. Chromatographic separations were conducted on a Shim-pack VP-ODS C18 column (250 × 4.6 mm i.d., 5 μm particle size; Shimadzu Corporation, Kyoto, Kyoto Pref., Japan), maintained at 40 °C. The mobile phase consisted of acidified water (0.2% acetic acid, solvent A) and acetonitrile (solvent B), delivered at a flow rate of 1.0 mL/min under gradient conditions as follows: 30–36% B (0–7 min), 36–38% B (7–17 min), 38–45% B (17–25 min), 45–55% B (25–29 min), 55–80% B (29–31 min), 80–100% B (31–32 min), followed by 100% B (32–39 min) and re-equilibration to initial conditions. Detection was performed at 280 nm, and the injection volume was 20 μL. Data acquisition and processing were carried out using LabSolutions^®^ software, version Lite Main.

BRP samples were analyzed using a Waters 2695 HPLC system coupled to a 2998 photodiode array detector (Waters Corporation, Milford, MA, USA). Chromatographic separations were achieved on an Ascentis Express C18 column (150 × 4.6 mm i.d., 2.7 μm; Supelco, Bellefonte, PA, USA). The mobile phase consisted of acidified water with formic acid (0.1%, *v*/*v*) and acetonitrile, applied under gradient elution conditions as follows: 20 → 50% B in 40 min, 50 → 100% B in 90 min, 100% B (isocratic) until 95 min, 100 → 20% B until 100 min, 20% B (isocratic) up to 105 min. The flow rate was set at 1.0 mL/min, and the detector was set to scan in the range of 220–600 nm.

The identification of compounds in both propolis samples was performed by comparison of retention times and UV spectral profiles with authentic reference compounds previously isolated and structurally characterized by our research group, together with previously reported chromatographic data.

### 2.2. Pathogens Used in the Study

The bacteria used in the antibacterial assays were obtained from the American Type Culture Collection (ATCC), except for the *Enterobacter cloacae* complex CDC 3430, a clinical isolate obtained from the Adolfo Lutz Institute (IAL), São Paulo, Brazil, collection (IAL 0124) [34]. The strains used were *Staphylococcus epidermidis* (ATCC 12228 and ATCC 14990), *Neisseria gonorrhoeae* (ATCC 43069), *Escherichia coli* (ATCC BAA-198), *E. cloacae* (IAL 124), *Staphylococcus aureus* (ATCC 29213), *Streptococcus pneumoniae* (ATCC 6305), *Listeria monocytogenes* (ATCC 15313), *Streptococcus agalactiae* (GBS, ATCC 13813), *Streptococcus pyogenes* (ATCC 19615), and *Enterococcus faecalis* (ATCC 29212 and ATCC 51299). Notably, according to the ATCC strain characterization, the panel included the multidrug-resistant, TEM-26 ESBL-producing *E. coli* ATCC BAA-198 and the vancomycin- and high-level aminoglycoside-resistant *E. faecalis* ATCC 51299 [35,36,37]. *Candida albicans* (ATCC 90028) was included as the fungal representative. The microorganisms are part of the collection of the Laboratory of Antimicrobial Testing (LEA) at the Federal University of Uberlândia (UFU) and were cryopreserved at −20 °C until the beginning of the experiments. For antiviral assays, the wild-type ZIKV isolate PE243, obtained from a patient in Brazil [38] was used and amplified and titrated as previously described [39].

### 2.3. Antimicrobial Activity

#### Minimum Inhibitory Concentration (MIC), Minimum Bactericidal Concentration (MBC), and Minimum Fungicidal Concentration (MFC)

The minimum inhibitory concentration (MIC) was defined as the lowest concentration capable of visibly inhibiting microbial growth. Assays were performed using the broth microdilution method in 96-well microplates (TPP, Trasadingen, Schaffhausen, Switzerland), according to the methodology recommended by the Clinical and Laboratory Standards Institute [40] with adaptations.

Briefly, 1 mg of each BRP and BGP-C extract was weighed and dissolved in 5% dimethyl sulfoxide (DMSO; Sigma-Aldrich, St. Louis, MO, USA), followed by dilution in Brain Heart Infusion broth (BHI; Kasvi, Pinhais, Paraná, Brazil), yielding final concentrations ranging from 0.195 to 400 µg/mL. For *S. pneumoniae* (ATCC 6305) and *L. monocytogenes* (ATCC 15313), BHI broth (Kasvi) was supplemented with 5% lysed horse blood. The inoculum was standardized to a McFarland standard of 0.5, as measured on a densitometer (Biomérieux, Marcy-l′Étoile, Lyon, France), and diluted to a bacterial concentration of 5 × 10^5^ CFU/mL in the wells. Gentamicin (Sigma-Aldrich) was used for technical control of the broth microdilution assay, according to CLSI recommendations, against *S. aureus* (ATCC 29213; expected MIC range: 0.12–1 µg/mL) and *E. coli* (ATCC 25922; expected MIC range: 0.25–1 µg/mL), at concentrations ranging from 0.0115 to 5.9 µg/mL. Additionally, the MICs of Gentamicin (Sigma-Aldrich) and Tetracycline (Sigma-Aldrich) were determined against the bacterial strains evaluated, and both antibiotics were included as positive controls. Tetracycline was tested at concentrations ranging from 0.0115 to 5.9 µg/mL. Sterility controls were performed for all broths and samples in the absence of bacterial inoculum. Growth controls consisted of wells containing only culture medium and inoculum. Additionally, solvent controls were included, containing culture medium, inoculum, and 5% DMSO (Sigma-Aldrich), to ensure that any observed antimicrobial effects were not attributable to the solvent. Microplates were incubated under aerobic conditions for 24 h at 37 °C; for assays with *S. pneumoniae* (ATCC 6305), incubation was performed in an incubator (PHC Corporation, Tokyo, Japan) under an atmosphere containing 10% CO_2_ for 20 h at 37 °C. After incubation, 30 µL of resazurin (Sigma-Aldrich) at a concentration of 0.02%, prepared in aqueous solution, was added to all wells, and plates were incubated again for 30 min. Metabolically active microorganisms reduce resazurin to resorufin, causing a color change from blue to pink, thereby allowing visual assessment of bacterial growth [41].

To evaluate antifungal activity against *C. albicans* (ATCC 90028), the MIC was determined by the broth microdilution method, following the same methodological principles described for bacteria, with adaptations recommended by the CLSI for yeasts [42]. BGP-C and BRP extracts were solubilized in 5% DMSO (Sigma-Aldrich) and diluted in Roswell Park Memorial Institute medium (RPMI 1640; Gibco Life Technologies, Thermo Fisher Scientific, Waltham, MA, USA) buffered with MOPS (3-(N-morpholino) propanesulfonic acid) (Sigma-Aldrich), yielding final concentrations ranging from 0.195 to 400 µg/mL. The fungal inoculum was standardized to a McFarland scale of 0.5, as measured on a densitometer (Biomérieux), and adjusted to a final concentration of 0.5 × 10^3^ CFU/mL in the wells. Microplates were incubated at 37 °C for 24 h. MIC reading was performed by adding resazurin (0.01%), followed by re-incubation for 4 h, with the MIC defined as the lowest concentration capable of preventing color change in the medium. Amphotericin B (Sigma-Aldrich) was used as a technical control for the broth microdilution assay, according to CLSI recommendations, against *C. krusei* (ATCC 6258; expected MIC range: 0.5–2 µg/mL) and *C. parapsilosis* (ATCC 22019; expected MIC range: 0.25–1 µg/mL).

To determine the minimum bactericidal concentration (MBC) and minimum fungicidal concentration (MFC), following microplate incubation and MIC determination, aliquots from each well were aspirated and plated onto appropriate agar media. For bacterial strains, aliquots were plated onto BHI agar (Kasvi). For *S. pneumoniae* (ATCC 6305) and *L. monocytogenes* (ATCC 15313), BHI agar (Kasvi) supplemented with 5% defibrinated horse blood was used. Plates were incubated under aerobic conditions for 24 h at 37 °C; for *S. pneumoniae* (ATCC 6305), incubation was performed in an incubator (PHC Corporation) under an atmosphere containing 10% CO_2_ for 20 h at 37 °C. For the fungal strain, aliquots were subcultured onto Sabouraud dextrose agar (SDA; Difco Laboratories, Detroit, MI, USA) and incubated at 37 °C for 24 h. The MBC and MFC were defined as the lowest concentrations at which no microbial growth was observed after subculturing. All assays were performed in triplicate.

### 2.4. Antibiofilm Activity

#### 2.4.1. Evaluation of Biofilm Biomass and Metabolic Activity

The evaluation of antibiofilm activity was performed using the sample selected after MIC testing and only extracts presenting MIC values ≤ 100 µg/mL were considered for subsequent assays [43]. Initially, the ability of the evaluated strains to form biofilms under in vitro conditions was determined as described by Stepanovic et al. (2007) [44].

For this purpose, microorganisms were incubated at concentrations of 10^6^, 10^7^, 10^8^, and 10^9^ CFU/mL in 96-well plates containing BHI broth (Kasvi) for 24, 48, and 72 h at 37 °C under aerobic conditions. For *S. pneumoniae* (ATCC 6305), BHI broth (Kasvi) supplemented with 5% lysed horse blood and 2% glucose (Êxodo Científica, Sumaré, São Paulo, Brazil) was used, with incubation for 24, 48, and 72 h at 37 °C in a CO_2_ incubator (PHC Corporation). After incubation, the contents of the wells were carefully removed, and the wells were washed three times with ultrapure distilled water to remove planktonic cells. The remaining biofilm was then fixed with methanol (Êxodo Científica, Sumaré/SP, Brazil) for 15 min, followed by staining with 2% crystal violet (Sigma-Aldrich) for 20 min. Subsequently, the wells were washed again with ultrapure distilled water to remove excess dye, and the adhered biofilm was solubilized with 200 µL of 33% acetic acid (Sigma-Aldrich) for 30 min [44,45]. Absorbance was measured using a microplate reader (Spectrostar Nano—BMG LABTECH GmbH, Ortenberg, Germany) at a wavelength of 595 nm. Biofilm formation was considered positive when absorbance was greater than or equal to 1, and the optimal concentration for biofilm formation was 10^8^ CFU/mL for all bacteria after 24 h.

To evaluate antibiofilm activity, as described by [45], bacterial suspensions were adjusted using a densitometer (Biomérieux) to yield a final inoculum concentration of 1.5 × 10^8^ CFU/mL in the wells. Subsequently, 100 µL of each inoculum was added to 96-well microplates (TPP) containing 100 µL of the propolis extract solutions, resulting in a final volume of 200 µL per well and final extract concentrations ranging from 0.195 to 400 µg/mL. Tetracycline (Sigma-Aldrich) was used as a positive control at concentrations ranging from 0.115 µg/mL to 59 µg/mL. Sterile broths were included as a negative control, while wells containing only bacteria without sample addition were used as growth controls. Microplates were incubated at 37 °C for 24 h; for *S. pneumoniae* (ATCC 6305), incubation was performed in an incubator with CO_2_ (PHC Corporation) for 20 h at 37 °C. In each assay, two microplates (TPP) were used, one for determination of biofilm biomass inhibition and the other for evaluation of metabolic activity. For biomass inhibition determination, after the incubation period, wells were washed, fixed, and stained as previously described. The minimum biofilm inhibitory concentration (MBIC_50_) was defined as the lowest sample concentration capable of reducing biofilm biomass by at least 50%. The percentage of inhibition was calculated by comparing the optical density (OD) of treated wells with that of untreated wells, according to the equation described by Wei et al., 2006 [46].

$$\mathrm{Inhibition}\;(\%)=1-\frac{\left(At595nm\right)}{\left(Ac595nm\right)}\times 100$$

where At595nm represents the absorbance of the treated well and Ac595nm represents the absorbance of the untreated well.

To evaluate the metabolic activity of biofilm cells, the XTT tetrazolium salt reduction assay was used, as described by Pierce et al. (2008, 2010) [47,48]. This assay is based on the conversion of the tetrazolium salt 2,3-bis(2-methoxy-4-nitro-5-sulfophenyl)-5-[carbonyl(phenylamino)]-2H-tetrazolium hydroxide (XTT; Sigma-Aldrich) into a formazan product by metabolically active cells, allowing indirect quantification of cell viability in biofilms. After the incubation period, the supernatant was carefully removed, and wells were washed with ultrapure distilled water to remove planktonic cells. Subsequently, 50 µL of XTT solution at 1 mg/mL in phosphate-buffered saline was added, together with 4 µL of menadione (Sigma-Aldrich). Plates were incubated again at 37 °C for 3 h. Metabolic activity was determined by measuring the absorbance of the formed product using a microplate reader (Spectrostar Nano) at a wavelength of 495 nm. The concentration capable of reducing biofilm metabolic activity by 50% was defined as IC_50_. Quantitative data obtained from the XTT reduction assay were analyzed using GraphPad Prism software, version 8.0.

#### 2.4.2. Analysis of Cell Viability in Biofilms Using Propidium Iodide (PI) and 4′,6′-Diamidino-2-phenylindole (DAPI)

Cell viability in biofilms was assessed by differential staining with propidium iodide (PI; Sigma-Aldrich) and 4′,6′-diamidino-2-phenylindole (DAPI; Thermo Fisher), as described by Williams et al. (1998) [49]. Differential DAPI/PI staining was used for the qualitative assessment of cell viability and membrane integrity within biofilms. DAPI (Thermo Fisher) binds to adenine–thymine-rich regions of DNA and was used as a counterstain to visualize bacterial cells, whereas PI (Sigma-Aldrich) preferentially enters cells with damaged membranes, intercalates into nucleic acids, and emits red fluorescence. Accordingly, increased PI (Sigma-Aldrich)-associated fluorescence was used as a qualitative indicator of compromised membrane integrity.

For this assay, biofilms were formed on glass coverslips (13 mm) placed in 24-well plates (TPP) and treated with BGP-C and BRP extracts at concentrations previously determined as MBIC_50_. Bacterial suspensions were standardized as previously described, added to the wells at a final concentration of 1 × 10^8^ CFU/mL, and incubated under the same conditions previously described for biofilm formation. After incubation, the contents of the wells were carefully aspirated using a micropipette, and the biofilms were gently washed three times with ultrapure distilled water to remove planktonic and non-adherent cells while preserving the sessile biofilm. Subsequently, 500 µL of DAPI solution (Thermo Fisher) at a concentration of 1 µg/mL was added to each well, followed by incubation for 10 min at room temperature under agitation and protected from light. The DAPI solution was then carefully aspirated, and 500 µL of PI solution (Sigma-Aldrich) at a concentration of 10 µg/mL was added to each well, followed by incubation for 30 min at room temperature without agitation and protected from light. After staining, the dye solutions were carefully aspirated, and the biofilms were washed with phosphate-buffered saline (PBS). Untreated biofilms were used as controls. Different regions of each coverslip were examined, and at least four fields per coverslip were captured using an EVOS M5000 Imaging System fluorescence microscope with a 40× objective. The DAPI and RFP fluorescence channels were used to detect DAPI-associated blue fluorescence and PI-associated red fluorescence, respectively. The assay was performed in three independent biological replicates.

#### 2.4.3. Biofilm Analysis by Scanning Electron Microscopy (SEM)

Morphological analysis of biofilm inhibition was performed by scanning electron microscopy, as described by Silva et al. (2024) [50], with modifications. For biofilm formation, bacteria were incubated in 24-well polystyrene plates (TPPs) containing sterilized polyvinyl chloride (PVC) discs with a diameter of 9 mm. Biofilms were treated with propolis extracts at concentrations corresponding to the previously determined MIC. All assays were performed in triplicate. Plates were incubated at 37 °C for 24 h; for *S. pneumoniae* (ATCC 6305), incubation was carried out under an atmosphere containing 10% CO_2_. After the incubation period, the supernatant was carefully removed, and the discs remained in the wells throughout all subsequent fixation and dehydration steps. Primary fixation was performed directly in the wells by adding a solution containing glutaraldehyde (Sigma-Aldrich) (2.5%) and paraformaldehyde (Sigma-Aldrich) (2 %) prepared in 0.15 M sodium cacodylate buffer (pH 7.0), followed by incubation for 2 h at room temperature. The fixative solution was then removed, and post-fixation was also carried out in the wells using a 1% osmium tetroxide solution (Sigma-Aldrich) for 2 h. After post-fixation, the discs were dehydrated directly in the wells through a graded ethanol (Êxodo científica) series at concentrations of 30%, 50%, 70%, 90%, and 100%, with 20 min intervals at each step. Only after completion of the dehydration step were the discs carefully removed from the plates and subjected to critical point drying (CPD) using liquid carbon dioxide in a Balzers CPD 030 apparatus. After drying, the discs were mounted on metal stubs and sputter-coated with gold. Samples were analyzed using a VEGA 3 LMU scanning electron microscope (TESCAN, Brno, Czech Republic) operating at magnifications of ×1000, ×5000, ×10,000, and ×20,000. Representative images of each experimental condition were selected based on structural integrity and biofilm representativeness.

### 2.5. In Vivo Assessment of Toxicity and Antimicrobial Activity in Caenorhabditis elegans

Before the infection assays, the toxicity of propolis extracts was evaluated in the mutant strain *Caenorhabditis elegans* AU37 [glp-4(bn2); sek-1(km4)] according to Singulani et al. (2017) [51]. Larvae were cultured on Nematode Growth Medium (NGM) plates previously seeded with *E. coli* OP50 and incubated at 16 °C for three days. After this period, nematodes were synchronized to the L4 stage by treatment with sodium hypochlorite and NaOH solution, followed by recovery of viable eggs. Subsequently, L4 larvae were washed with M9 buffer and a 20 μL aliquot of the larval suspension was dispensed into each well of a 96-well flat-bottom microplate (TPP) containing 80 μL of BHI broth (Kasvi) supplemented with streptomycin (200 μg/mL; Sigma-Aldrich, Barueri, Brazil), ampicillin (200 μg/mL; Sigma-Aldrich, Barueri, Brazil), kanamycin (90 μg/mL; Sigma-Aldrich, Barueri, Brazil) and the propolis extracts at final concentrations ranging from 187.5 to 6000 μg/mL, resulting in a final volume of 100 μL per well. The plates were incubated at 25 °C for four days, and larval survival was monitored daily by counting live and dead individuals. Viability was assessed based on nematode motility and morphology. The concentrations selected for the infection assays were defined based on the toxicity assay performed in the present study. Additionally, previous work from our group demonstrated low toxicity of BRP in *C. elegans*, reporting an LC_50_ of 1500 μg/mL [52].

The antimicrobial activity of the extracts was evaluated using the same *C. elegans* AU37 strain, following methodologies adapted from Singulani et al. (2017) [51] and Moy et al. (2009) [53]. The nematodes were cultured and synchronized to the L4 stage as previously described. After synchronization, the larvae were transferred to BHI agar (Kasvi) plates inoculated with the evaluated bacteria and incubated for 12 h to establish infection. The *S. pneumoniae* (ATCC 6305) and *L. monocytogenes* (ATCC 15313) strains were cultured on BHI agar (Kasvi) supplemented with defibrinated horse blood, while the other strains were cultured on BHI agar (Kasvi) without supplementation. After the infection period, the larvae were washed with M9 buffer and centrifuged to remove bacteria adhering to the external surface of the nematodes. The larval suspension was transferred to microplates prepared as described for the toxicity assay, and propolis extracts were added at MIC and 2 × MIC concentrations. The plates were incubated at 25 °C for four days, and larval survival was monitored daily. Viability was determined based on nematode motility and morphology. On the fourth day post-infection, the nematodes were additionally stained with erythrosine, used as an auxiliary method for confirming mortality. Infected and untreated larvae were used as a negative control, while infected larvae treated with tetracycline at the MIC were used as a positive control. A group of uninfected larvae was included as a baseline viability control. All experiments were performed in duplicate.

### 2.6. Antiviral Activity

#### 2.6.1. Cell Culture

Vero E6 cells (ATCC CRL-1586), derived from normal adult African green monkey kidney tissue (*Chlorocebus sabaeus*), and human villous trophoblast BeWo cells (ATCC CCL-98) were used in this study. Cell lines were cultured in Dulbecco’s Modified Eagle Medium (DMEM; Sigma–Aldrich) supplemented with 100 U/mL penicillin (Gibco Life Technologies), 100 µg/mL streptomycin (Gibco Life Technologies), 1% (*v*/*v*) non-essential amino acids (Gibco Life Technologies), and 10% (*v*/*v*) fetal bovine serum (FBS; HyClone Laboratories, Logan, UT, USA). Cell cultures were maintained at 37 °C in a CO_2_ incubator (PHC Corporation) under 5% CO_2_.

All ZIKVPE243 infection assays were conducted in a BSL-2 laboratory under authorization number CBQ: 163/02 and SEI process no. 01.245.006267/2022–14, issued by CTNBio—“Comissão Técnica Nacional de Biossegurança do Brazil”.

#### 2.6.2. Viral Amplification and Titration in Vero E6 Cells

The wild-type Zika virus isolate (ZIKVPE243) [38], was amplified using Vero E6 cells (ATCC CRL-1586) infected in 75 cm2 flasks (Kasvi) for 3 days at 37 °C in a CO_2_ incubator (PHC Corporation) under 5% CO_2_. Viral titers were determined by a plaque formation assay, in which Vero E6 cells were seeded in 24-well plates at a density of 8 × 10^4^ cells/well 24 h prior to infection. Cells were then infected with serial tenfold dilutions of ZIKVPE243 and incubated at 37 °C. After 1 h, the viral inoculum was removed and replaced with fresh medium supplemented with 2% fetal bovine serum (FBS; HyClone) and 1% carboxymethylcellulose (CMC; Sigma–Aldrich, Barueri, Brazil). Infected cells were incubated for 72 h at 37 °C in a CO_2_ incubator (PHC Corporation) with 5% CO_2_. Subsequently, the medium was removed, and cells were fixed with 4% formaldehyde (Synth, Diadema, São Paulo, Brazil) for 30 min and stained with 0.5% crystal violet (Sigma-Aldrich, Barueri, Brazil) for 15 min. Viral plaques were counted to determine the ZIKVPE243 titer, expressed as plaque-forming units per milliliter (PFU/mL).

#### 2.6.3. Cell Viability and Antiviral Assay in Vero E6 Cells

Cell viability was measured by the MTT method [3-(4,5-dimethylthiazol-2-yl)-2,5-diphenyl tetrazolium bromide] (INLAB, Diadema, São Paulo, Brazil). Vero E6 cells were seeded in 96-well plates at a density of 5 × 10^3^ cells per well and incubated overnight at 37 °C in a CO_2_ incubator (PHC Corporation) with 5% CO_2_. After incubation, the culture medium was replaced with medium containing propolis samples at concentrations of 50, 10, and 2 µg/mL, and cells were incubated for 72 h at 37 °C. In BeWo cells, seeding was performed at a density of 1 × 10^4^ cells/well and cells were treated with BGP-C or BRP at concentrations ranging from 100 to 0.2 µg/mL. Medium was then removed, and an MTT (INLAB) solution at a final concentration of 1 mg/mL was added to each well, followed by incubation for 30 min in a 37 °C incubator (PHC Corporation). After this period, the solution was removed and 100 µL of DMSO (Sigma-Aldrich, Barueri, Brazil) was added to each well to solubilize the formazan crystals. Absorbance was measured as optical density (OD) at 560 nm using a GloMax microplate reader (Promega Corporation, Madison, WI, USA). Cell viability was calculated according to the equation (T/C) × 100%, where T represents the mean optical density of the treated group and C represents the mean optical density of the vehicle control group. In BeWo cells, the 50% cytotoxic concentration (CC_50_) was determined by nonlinear regression using GraphPad Prism software version 8.0.

To evaluate the antiviral activity of each propolis sample, Vero E6 cells were seeded at a density of 5 × 10^3^ cells per well in 96-well plates for 24 h and infected with ZIKV_PE243_ at a multiplicity of infection (MOI) of 0.01 in the presence or absence of BGP-C or BRP, both at the established non-cytotoxic concentrations. In BeWo cells, seeding was performed at a density of 1 × 10^4^ cells/well in 96-well plates for 24 h and subsequently infected with ZIKV_PE243_ at an MOI of 0.01 in the presence of each compound, following the same concentration range from the BeWo viability assay (100 to 0.2 µg/mL). Cells were then fixed with 4% formaldehyde (Synth), washed with PBS, and incubated with blocking buffer (BB) containing 0.1% Triton X-100 (Vetec Química Fina Ltd., Rio de Janeiro, Brazil), 0.2% bovine serum albumin (BSA), and PBS for 30 min, followed by the immunofluorescence assay as previously described [39]. Briefly, after the blocking step, cells were incubated for 1 h with a primary rabbit polyclonal antibody directed against the ZIKV NS3 protein, diluted in BB. Cells were then washed and incubated for 1 h with Alexa Fluor 488-conjugated anti-rabbit IgG (Invitrogen, Carlsbad, CA, USA) as a secondary antibody, also diluted in BB. Images were acquired by fluorescence microscopy using the EVOS Cell Imaging System (Thermo Fisher Scientific), and each focus of infection, defined as a cluster of NS3-positive cells, was counted and expressed as focus-forming units (FFU). Antiviral activity was calculated according to the equation (T/C) × 100%, where T and C represent the mean values of the treated group and the vehicle control, respectively. The 50% effective concentration (EC_50_) in BeWo cells was determined by nonlinear regression using GraphPad Prism software version 8.0 [39]. CC_50_ and EC_50_ values were used to calculate the selectivity index (SI = CC_50_/EC_50_). In all ZIKV assays, infected cells treated with the vehicle (0.1% DMSO) were used as an untreated control.

### 2.7. Statistical Analyses

GraphPad Prism software (version 8.0) was used for quantitative data analysis. For biofilm assays, the 50% inhibitory concentration of metabolic activity (IC_50_) was determined from dose–response curves obtained in the XTT reduction assay.

For *C. elegans* assays, survival curves were estimated using the Kaplan–Meier method and compared using the log-rank (Mantel–Cox) test. Cox proportional hazard regression was used to estimate hazard ratios (HRs), and the proportional hazards assumption was not satisfied; restricted mean survival time (RMST) over the four-day follow-up period was used as the primary measure of treatment effect. Differences in RMST (Δ RMST) were estimated between treated and untreated infected groups and between treatment groups and the tetracycline-treated group, with adjustment for multiple comparisons using the Holm method. Differences in virulence among bacterial strains were additionally evaluated using Kaplan–Meier analysis and Cox proportional hazards regression, with pairwise comparisons adjusted using the Tukey method. Survival analyses were performed in R using the survival, survminer, emmeans, ggplot2, and flextable packages.

In antiviral assays, dose–response curves were fitted by nonlinear regression using a log inhibitor versus response model with variable slope, applying a four-parameter logistic model to determine the 50% cytotoxic concentration (CC_50_) and the 50% effective concentration (EC_50_). The selectivity index (SI) was calculated as the ratio of CC_50_ to EC_50_. For antiviral assays in Vero E6 cells, data were initially assessed for normal distribution, and comparisons between treatments and the vehicle control were performed using two-way analysis of variance (ANOVA), with *p* < 0.05 considered statistically significant.

## 3. Results

### 3.1. Chemical Characterization of Propolis Samples

**Figure 1:**
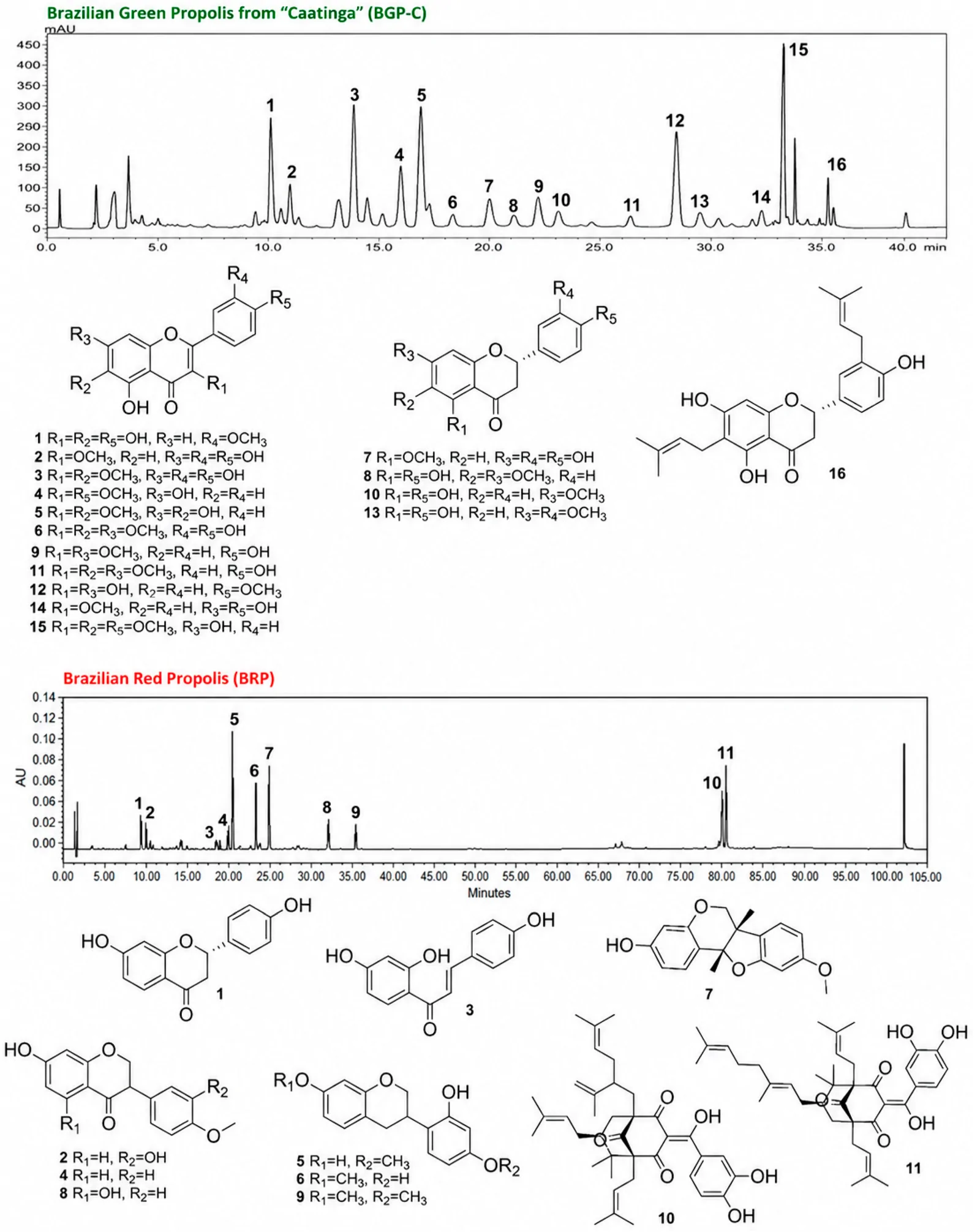
HPLC chromatogram of the Brazilian “Caatinga” green propolis (λ = 286 nm) and red propolis (λ = 275 nm). Peak assignments correspond to the compounds indicated in the figure.

The chemical profiles of BGP-C and BRP were investigated by HPLC-DAD analysis, revealing distinct chromatographic patterns that reflect their different botanical origins (Figure 1).

The chromatographic profile of BGP-C was characterized by a predominance of flavonoid derivatives, indicating a complex mixture of structurally related phenolic compounds. Based on retention time and UV spectral comparisons with previously reported data, sixteen flavonoids were identified, including representatives of flavonols, flavones, and flavanones. The identified compounds comprised tamarixetin (**1**), quercetin 3-methyl ether (**2**), axillarin (**3**), ermanin (**4**), viscosin (**5**), quercetagetin 3,6,7-trimethyl ether (**6**), eriodictyol 5-O-methyl ether (**7**), 5,4′-dihydroxy-6,7-dimethoxyflavanone (**8**), kumatakenin (**9**), sakuranetin (**10**), penduletin (**11**), kaempferide (**12**), eriodictyol-7,3′-methyl ether (**13**), isokaempferide (**14**), santin (**15**), and macarangaflavanone B (**16**). This flavonoid-rich composition is consistent with propolis derived from *M. tenuiflora* and reinforces its characteristic phytochemical signature.

BRP exhibited a different chromatographic profile, characterized by a broader distribution of peaks and increased chemical diversity. The HPLC-DAD analysis revealed the presence of different classes of secondary metabolites, including flavonoids, isoflavonoids, chalcones, isoflavanes, pterocarpans, and polyprenylated benzophenones. Based on chromatographic and spectral comparisons with literature data, the main constituents were assigned as liquiritigenin (**1**), calycosin (**2**), isoliquiritigenin (**3**), formononetin (**4**), vestitol (**5**), neovestitol (**6**), medicarpin (**7**), biochanin A (**8**), and 7-O-methylvestitol (**9**), along with polyprenylated benzophenone derivatives such as guttiferone E, xanthochymol, and oblongifolin B.

The BRP and BGP-C materials evaluated in the present study have previously been subjected to extensive phytochemical characterization and quantitative analysis by our research group. For BRP, a validated RP-HPLC-DAD method was previously applied to this sample and demonstrated vestitol (2.140–19.455% g/100 g of dried raw material), medicarpin (1.493–14.611%), and neovestitol (0.964–6.894%) as major quantified constituents. Other quantified markers included liquiritigenin (0.179–2.069%), calycosin (0.056–0.573%), isoliquiritigenin (0.210–2.409%), formononetin (0.238–2.640%), biochanin A (0.034–0.376%), and guttiferone E/xanthochymol (0.533–3.833%) [27]. For BGP-C, quantitative characterization of the same propolis material was also previously performed using a validated RP-HPLC-DAD method [31]. Among the quantified flavonoids, kaempferide was the predominant constituent (43.72 ± 1.81 mg/g of crude extract), followed by santin (11.73 ± 0.15 mg/g), viscosine (11.18 ± 0.17 mg/g), axillarin (10.18 ± 0.15 mg/g), tamarixetin (7.54 ± 0.78 mg/g), ermanin (6.09 ± 0.08 mg/g), kumatakenin (5.43 ± 0.11 mg/g), and eriodictyol-5-O-methyl ether (5.18 ± 0.06 mg/g). These quantitative data complement the chromatographic characterization and provide a more comprehensive description of the distinct phytochemical profiles of BRP and BGP-C.

### 3.2. Antibacterial Activity Evaluation

The MIC and MBC/MFC results of the propolis extracts against the bacteria and fungi included in the study are presented in Table 1. BGP-C and BRP extracts showed MIC values ranging from 6.25 to 400 µg/mL and MBC/MFC values from 25 to 400 µg/mL, with greater activity against Gram-positive bacteria.

Overall, the extracts exhibited predominantly bacteriostatic activity. A bactericidal effect was observed only for *S. pneumoniae* (ATCC 6305) and *S. agalactiae* (ATCC 13813) treated with BRP, for which MIC and MBC values were identical (50 µg/mL). For *C. albicans* (ATCC 90028), both extracts showed coincident MIC and MFC values (200 µg/mL), characterizing a fungicidal effect.

The BGP-C extract showed the lowest MIC values against *S. pneumoniae* (ATCC 6305) and *S. agalactiae* (ATCC 13813), both with MICs of 6.25 µg/mL and MBCs of 25 µg/mL. For *L. monocytogenes* (ATCC 15313), MIC and MBC values of 50 µg/mL and 200 µg/mL, respectively, were observed. In contrast, *S. epidermidis* (ATCC 14990) exhibited an MIC of 100 µg/mL and an MBC > 400 µg/mL, indicating a bacteriostatic effect.

The MIC values of gentamicin and tetracycline against the bacterial strains are presented in Supplementary Material Table S1.

### 3.3. Antibiofilm Activity Evaluation

#### 3.3.1. Effects of Propolis Extracts on Biofilm Biomass and Metabolic Activity

**Figure 2:**
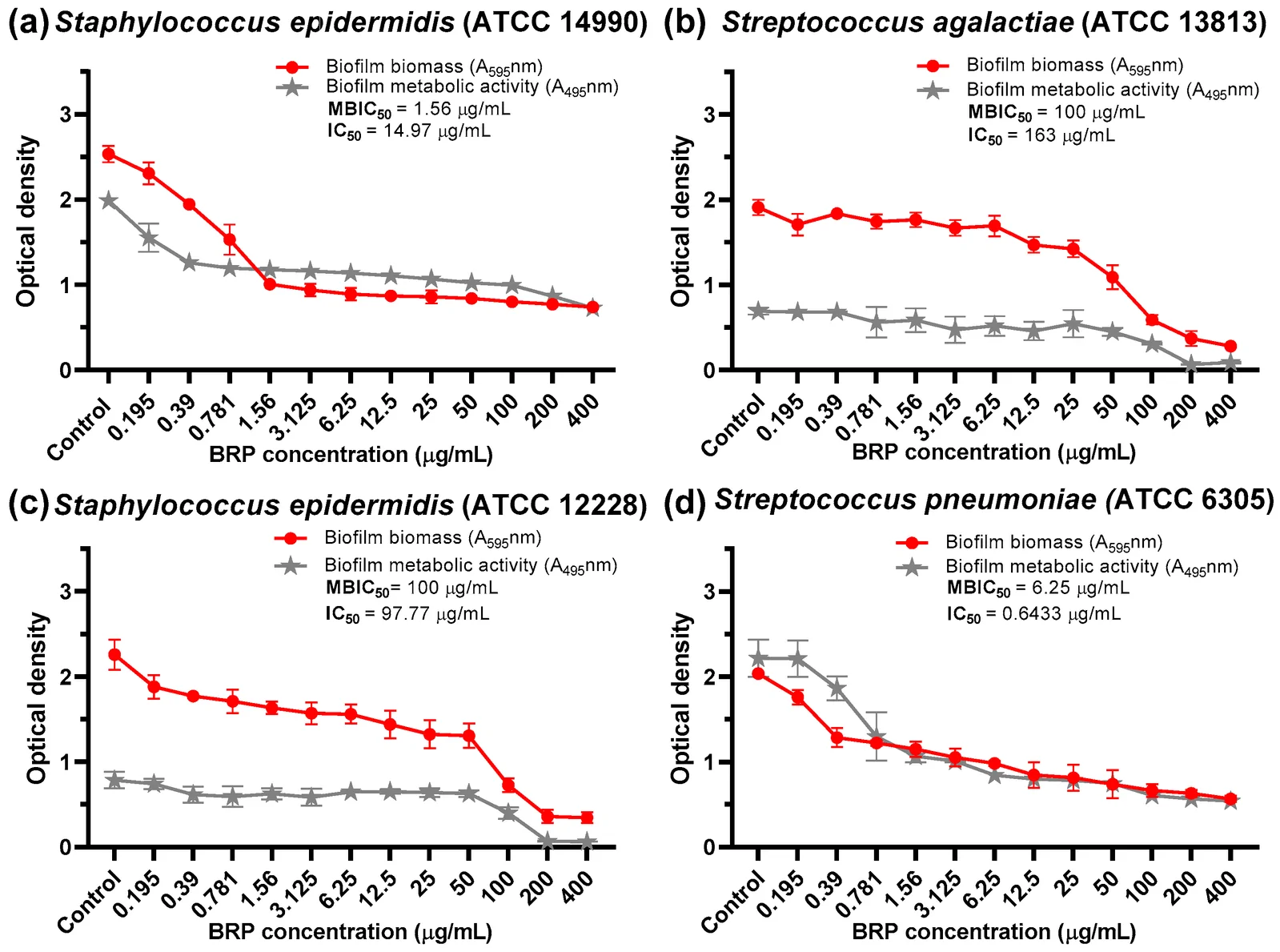
Antibiofilm activity of Brazilian red propolis (BRP) against monospecies biofilms formed by selected ATCC strains. Biofilm biomass and the metabolic activity of adhered cells were evaluated. (**a**) *Staphylococcus epidermidis* (ATCC 14990), (**b**) *Streptococcus agalactiae* (ATCC 13813), (**c**) *Staphylococcus epidermidis* (ATCC 12228), and (**d**) *Streptococcus pneumoniae* (ATCC 6305). Red circles represent biofilm biomass, and gray stars represent the metabolic activity of adhered cells. The minimum biofilm inhibitory concentration (MBIC_50_) was defined as the lowest extract concentration capable of reducing biofilm biomass by at least 50%. The concentration required to reduce the metabolic activity of adhered cells by 50% was defined as IC_50_.

**Figure 3:**
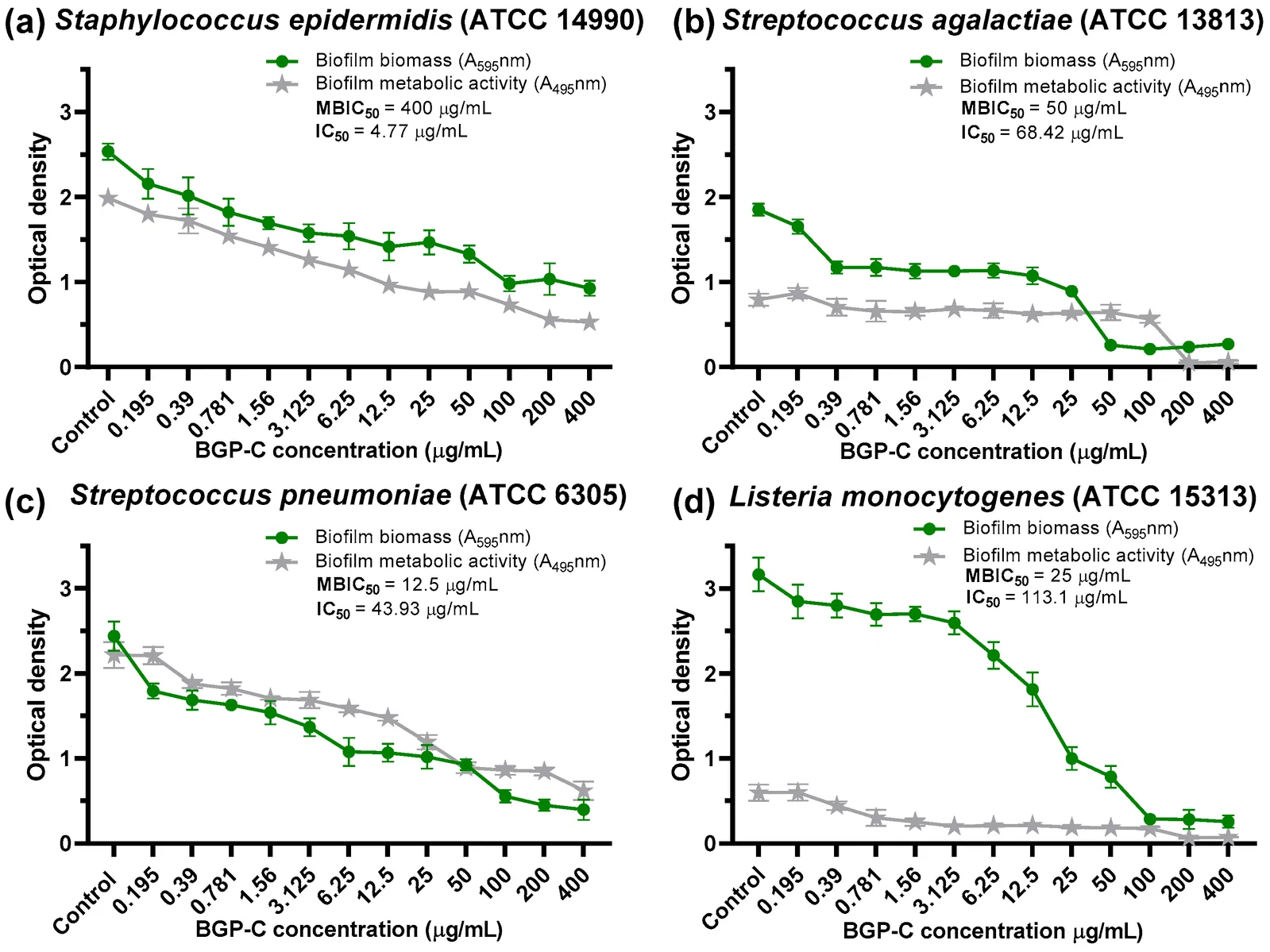
Antibiofilm activity of Brazilian green propolis from the “Caatinga” biome (BGP-C) against monospecies biofilms formed by selected ATCC strains. Biofilm biomass and the metabolic activity of adhered cells were evaluated. (**a**) *Staphylococcus epidermidis* (ATCC 14990), (**b**) *Streptococcus agalactiae* (ATCC 13813), (**c**) *Streptococcus pneumoniae* (ATCC 6305), and (**d**) *Listeria monocytogenes* (ATCC 15313). Green circles represent biofilm biomass, and gray stars represent the metabolic activity of adhered cells. The minimum biofilm inhibitory concentration (MBIC_50_) was defined as the lowest extract concentration capable of reducing biofilm biomass by at least 50%. The concentration required to reduce the metabolic activity of adhered cells by 50% was defined as IC_50_.

The antibiofilm activity of BGP-C and BRP was evaluated only against bacterial strains for which each extract exhibited MIC values ≤ 100 µg/mL. BRP was evaluated against *S. epidermidis* (ATCC 12228 and ATCC 14990), *S. pneumoniae* (ATCC 6305), and *S. agalactiae* (ATCC 13813), and the results are presented in Figure 2. BGP-C was evaluated against *S. epidermidis* (ATCC 14990), *S. pneumoniae* (ATCC 6305), *S. agalactiae* (ATCC 13813), and *L. monocytogenes* (ATCC 15313), and the results are presented in Figure 3. Both propolis extracts significantly reduced biofilm biomass compared with the untreated control. For BRP, MBIC_50_ values ranged from 1.56 to 100 µg/mL, and IC_50_ values ranged from 0.64 to 163 µg/mL. For BGP-C, MBIC_50_ values ranged from 12.5 to 400 µg/mL, and IC_50_ values ranged from 4.77 to 113.1 µg/mL.

For the BRP extract, the highest antibiofilm activity was observed against *S. pneumoniae* (ATCC 6305), with an IC_50_ of 0.64 µg/mL and an MBIC_50_ of 6.25 µg/mL. *S. epidermidis* showed a strain-dependent response, with greater susceptibility of ATCC 14990 (MBIC_50_ = 1.56 µg/mL; IC_50_ = 14.97 µg/mL) compared with ATCC 12228 (MBIC_50_ = 100 µg/mL; IC_50_ = 97.77 µg/mL). In contrast, *S. agalactiae* (ATCC 13813) exhibited lower relative efficacy, with an IC_50_ of 163 µg/mL and an MBIC_50_ of 100 µg/mL (Figure 2).

For the BGP-C extract, greater metabolic interference was observed in *S. epidermidis* (ATCC 14990), with an IC_50_ of 4.77 µg/mL, although biomass reduction required higher concentrations (MBIC_50_ = 400 µg/mL). Among the streptococci, *S. pneumoniae* (ATCC 6305) showed the highest efficacy (IC_50_ = 43.93 µg/mL; MBIC_50_ = 12.5 µg/mL), followed *by S. agalactiae* (ATCC 13813) (IC_50_ = 68.42 µg/mL; MBIC_50_ = 50 µg/mL), whereas *L. monocytogenes* (ATCC 15313) required higher concentrations for metabolic reduction (IC_50_ = 113.1 µg/mL), despite exhibiting a relatively low MBIC_50_ (25 µg/mL) (Figure 3). Regarding the positive control (Tetracycline), MBIC_50_ values for monospecies biofilms ranged from 0.092 to 0.737 µg/mL (Supplementary Material, Figure S1). IC_50_ values ranged from 0.027 to 0.300 µg/mL, depending on the strain evaluated.

#### 3.3.2. Biofilm Cell Viability

**Figure 4:**
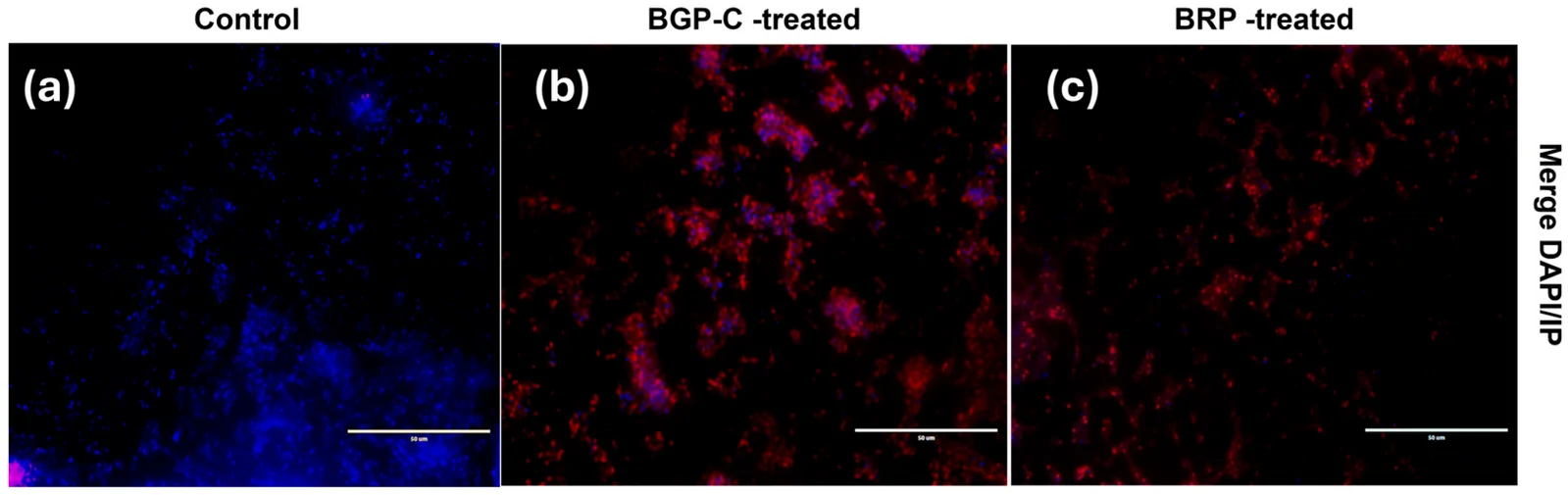
Fluorescence micrographs (DAPI/PI) of biofilms formed by the representative strain *S. epidermidis* (ATCC 14990). DAPI (4′,6-diamidino-2-phenylindole; blue) stained bacterial DNA, whereas propidium iodide (PI; red) indicated cells with compromised membrane integrity. Images show the untreated biofilm control and biofilms treated with Brazilian green propolis from the “Caatinga” biome (BGP-C) or Brazilian red propolis (BRP) at their respective minimum biofilm inhibitory concentrations (MBIC_50_). (**a**) Untreated biofilm control, (**b**) biofilm treated with BGP-C and (**c**) biofilm treated with BRP. The images are representative of three independent biological replicates. Scale bars represent 50 µm.

The viability of biofilm cells was qualitatively analyzed by fluorescent staining with DAPI/PI. The results shown in Figure 4 correspond to *S. epidermidis* (ATCC 14990), selected as the representative strain. Images related to the other evaluated strains are available in Supplementary Material (Figures S2 and S3). The control group showed predominantly blue fluorescence and low PI-associated red fluorescence. In contrast, biofilms treated with BGP-C and BRP extracts, both at the MBIC_50_ concentration, showed visually increased red fluorescence compared with the control, suggesting reduced cell viability and compromised membrane integrity. This qualitative pattern was consistently observed across the experimental replicates and analyzed strains.

#### 3.3.3. Scanning Electron Microscopy Images

**Figure 5:**
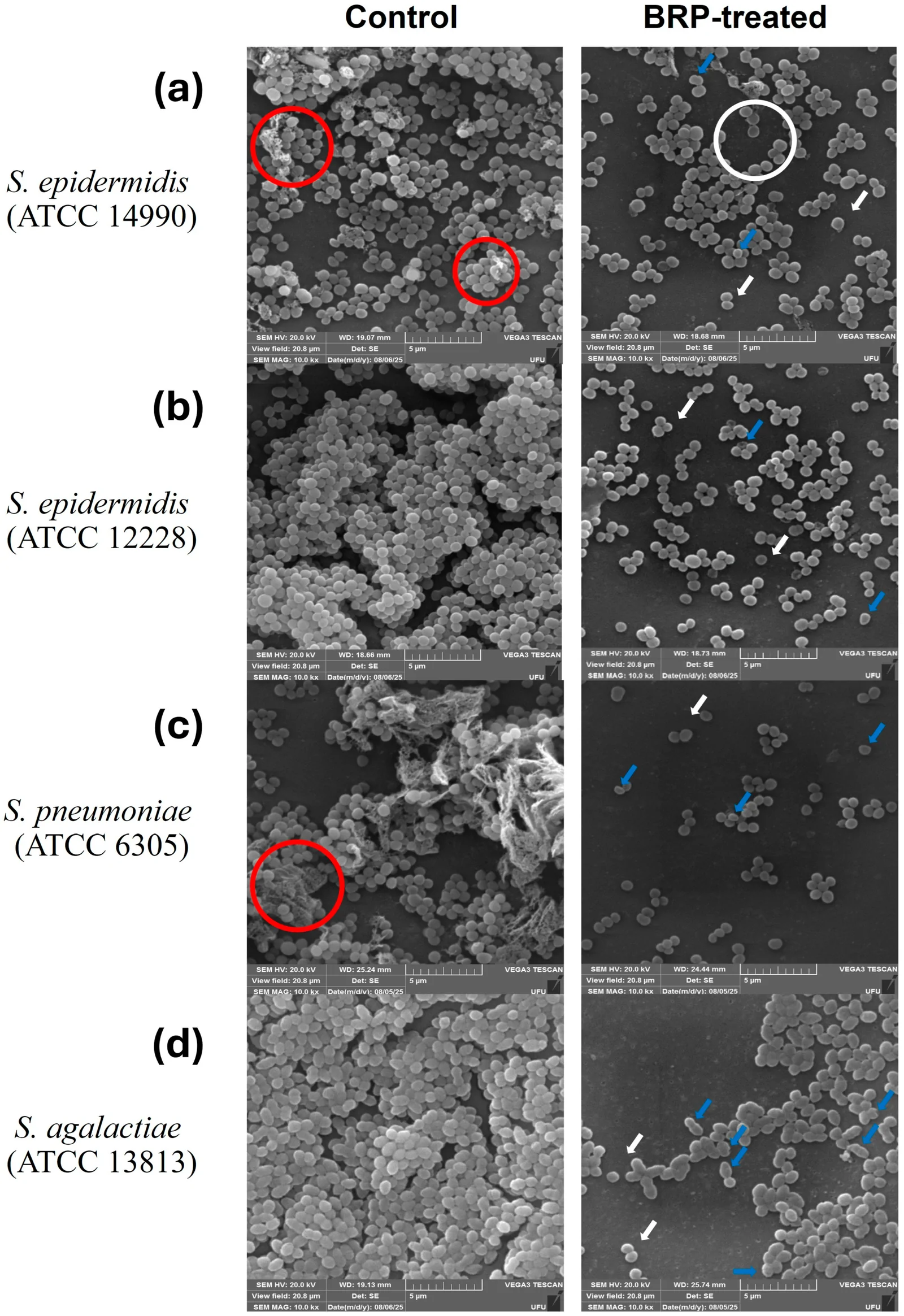
Scanning electron microscopy (SEM) micrographs of monospecies biofilms treated with Brazilian red propolis (BRP), obtained at 10,000× magnification. (**a**) *S. epidermidis* (ATCC 14990), (**b**) *S. epidermidis* (ATCC 12228), (**c**) *S. pneumoniae* (ATCC 6305), and (**d**) *S. agalactiae* (ATCC 13813). Red circles indicate the extracellular biofilm matrix, white arrows indicate dispersed bacterial cells, blue arrows indicate cellular morphological alterations, and white circles indicate reduction extracellular biofilm matrix.

SEM micrographs revealed consistent morphological differences between the control groups and those treated with propolis extracts, characterized by a reduction in the density of cells adhered to the surface, a decrease in the extracellular biofilm matrix, and cellular morphological alterations. The images shown are representative of the observed findings and were obtained at a magnification of 10,000×. In Figure 5, referring to biofilms treated with BRP, a reduction in the extracellular biofilm matrix (white circle) and a decrease in bacterial aggregates are observed, with a predominance of dispersed cells (white arrows), as exemplified in *S. epidermidis* (ATCC 14990) (Figure 5a) and *S. pneumoniae* (ATCC 6305) (Figure 5c). Cellular morphological alterations, indicated by blue arrows, are also evident in the treated groups, including biofilms produced by *S. epidermidis* (ATCC 12228) (Figure 5b) and *S. agalactiae* (ATCC 13813) (Figure 5d).

**Figure 6:**
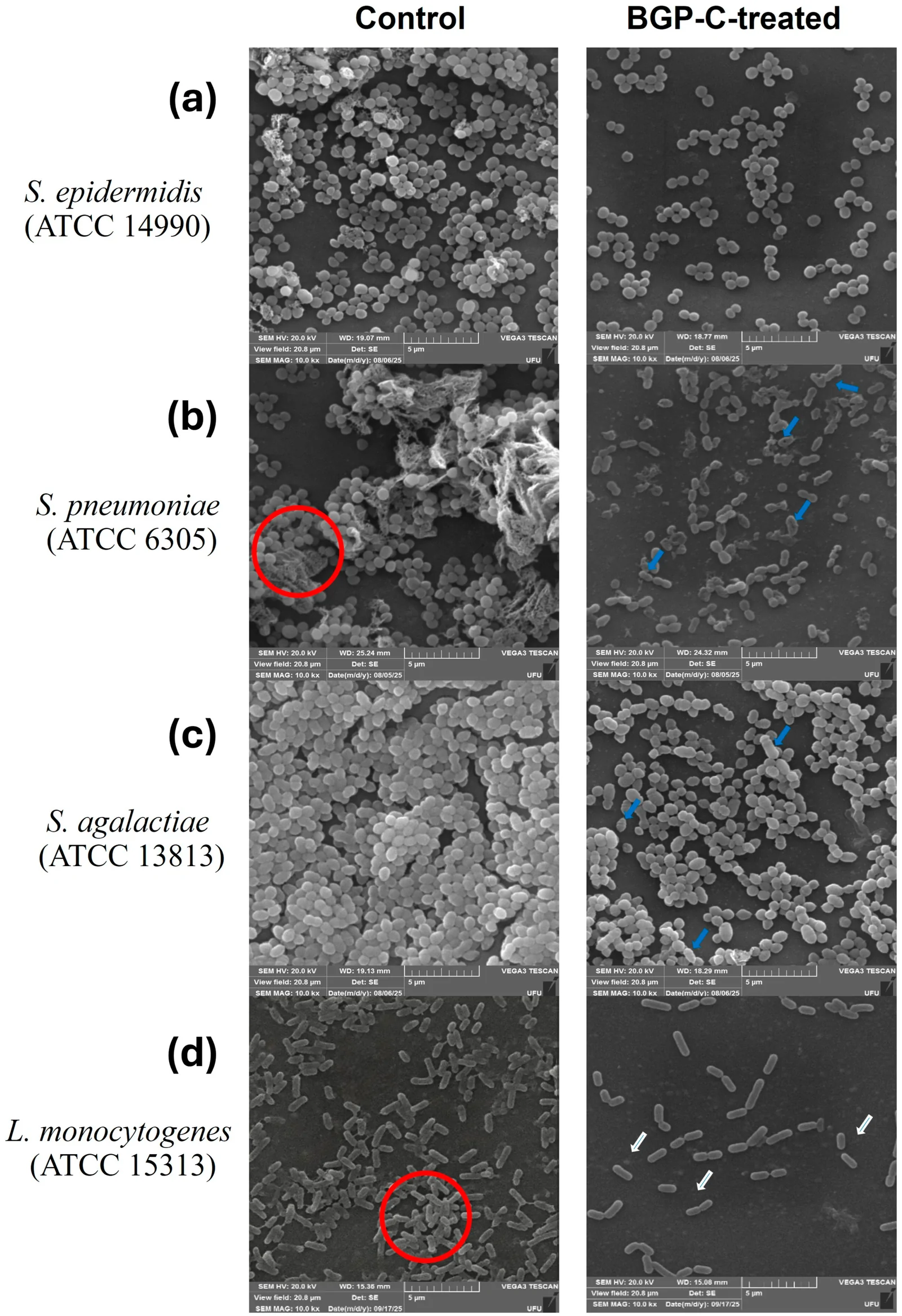
Scanning electron microscopy (SEM) micrographs of monospecies biofilms treated with Brazilian green propolis from the “Caatinga” biome (BGP-C), obtained at 10,000× magnification. (**a**) *S. epidermidis* (ATCC 14990), (**b**) *S. pneumoniae* (ATCC 6305), (**c**) *S. agalactiae* (ATCC 13813), and (**d**) *L. monocytogenes* (ATCC 15313). Red circles indicate the extracellular biofilm matrix, blue arrows indicate cellular morphological alterations, and white arrows indicate dispersed bacterial cells.

In Figure 6, corresponding to biofilms treated with BGP-C, a visual reduction in cell density and extracellular biofilm matrix is observed in *S. epidermidis* (ATCC 14990) (Figure 6a) compared with the control, with more pronounced cellular morphological alterations highlighted by blue arrows, as seen in *S. pneumoniae* (ATCC 6305) (Figure 6b) and *S. agalactiae* (ATCC 13813) (Figure 6c) as well as a reduction in cell density and a predominance of dispersed cells, indicated by white arrows, in biofilms produced by *L. monocytogenes* (ATCC 15313) (Figure 6d).

### 3.4. Effects of Propolis Extracts on Larval Survival and Bacterial Infection in Caenorhabditis elegans

**Figure 7:**
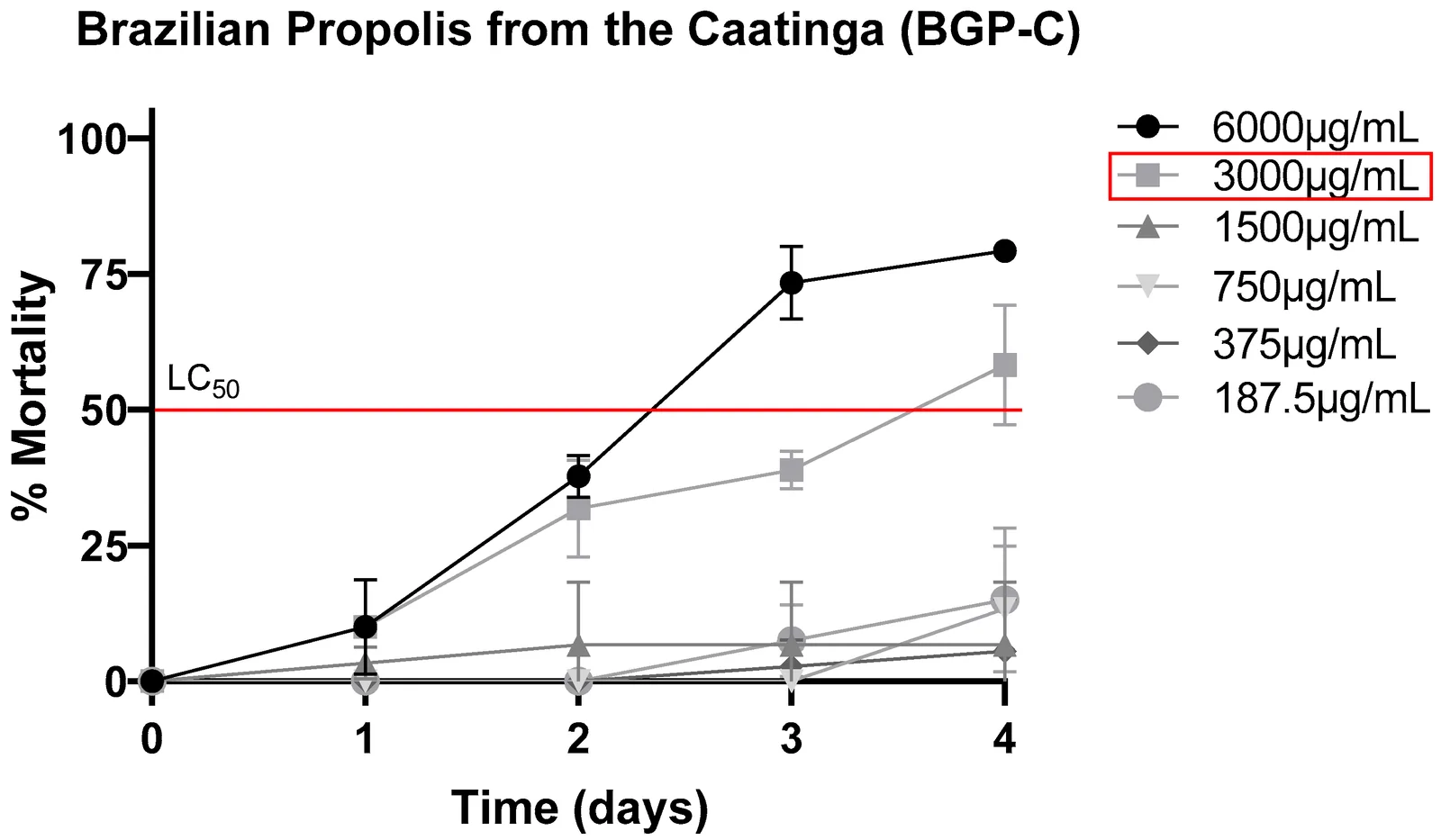
Toxicity of Brazilian propolis from the “Caatinga” (BGP-C) in *C. elegans* AU37. Larval mortality was monitored for four days at different BGP-C concentrations. The horizontal red line indicates 50% mortality (LC_50_), while the red box highlights 3000 µg/mL, the lowest tested concentration that resulted in >50% larval mortality.

Before evaluating the antimicrobial activity of BGP-C in the infection model, its toxicity was assessed in *C. elegans* AU37 larvae. On the fourth day of exposure, 3000 μg/mL was the lowest concentration resulting in >50% larval mortality, while lower concentrations showed low toxicity. These findings supported the concentrations selected for subsequent infection assays. (Figure 7).

**Figure 8:**
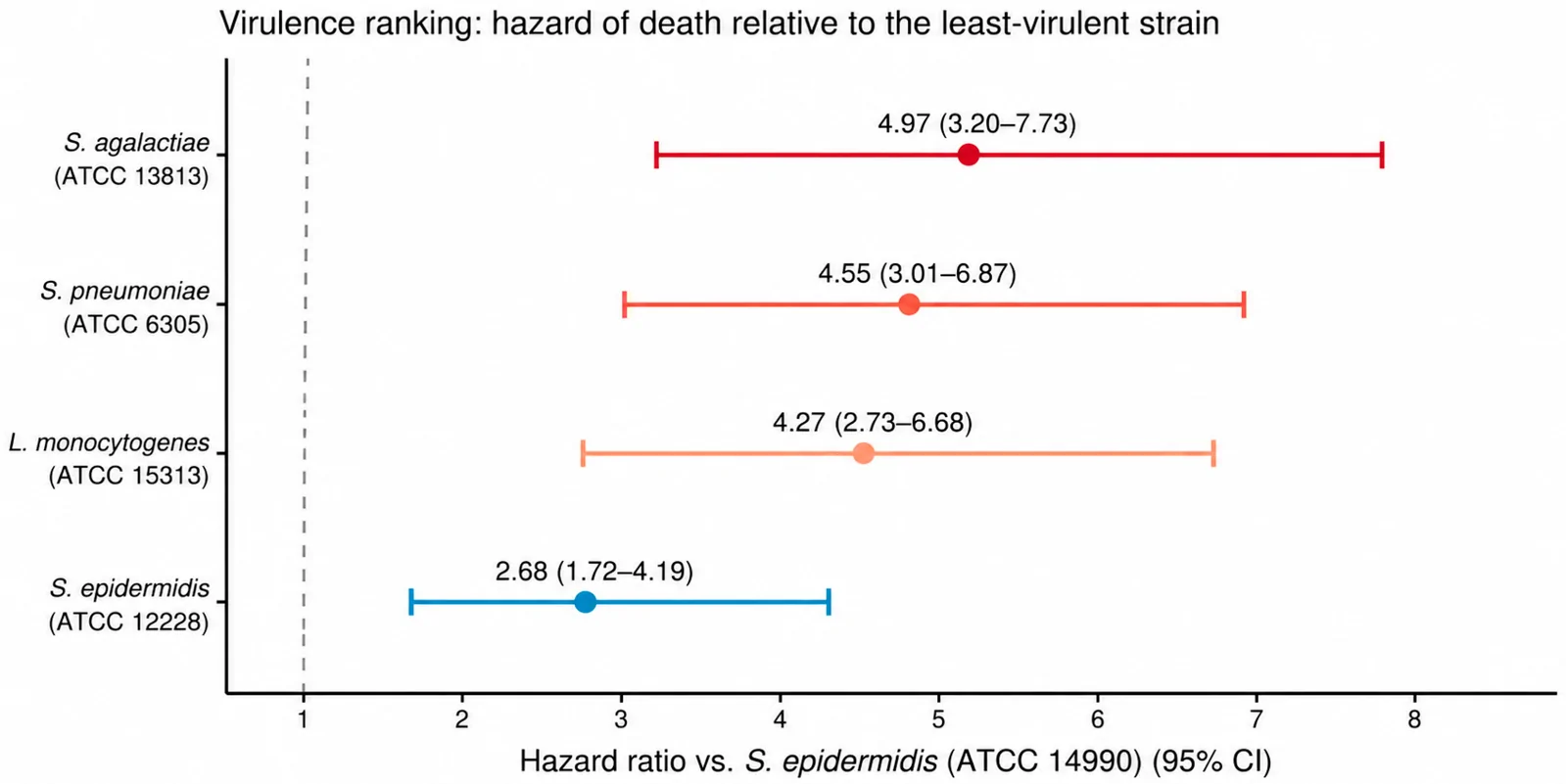
Relative virulence of bacterial strains in *C. elegans* AU37. Hazard ratios (HR) and 95% confidence intervals were estimated by Cox regression using *S. epidermidis* (ATCC 14990) as the reference strain. The dashed vertical line at HR = 1 indicates no difference in the hazard of death relative to the reference strain.

Considering the potential impact of bacterial virulence on treatment response, survival of *C. elegans* larvae infected with the different bacterial strains was first evaluated. Survival differed significantly among *C. elegans* larvae infected with the five bacterial strains (log-rank χ^2^ = 67.0, df = 4, *p* < 0.0001; Supplementary Material Figure S4). *S. agalactiae* (ATCC 13813), *S. pneumoniae* (ATCC 6305), and *L. monocytogenes* (ATCC 15313) showed the highest virulence, with a median survival of 2 days and complete mortality by day 4. In comparison, *S. epidermidis* (ATCC 12228) showed intermediate virulence, whereas *S. epidermidis* (ATCC 14990) resulted in the lowest overall mortality. Relative to *S. epidermidis* (ATCC 14990), the hazard of death was 4.97-fold higher for *S. agalactiae* (ATCC 13813), 4.55-fold for *S. pneumoniae* (ATCC 6305), 4.27-fold for *L. monocytogenes* (ATCC 15313), and 2.68-fold for *S. epidermidis* (ATCC 12228) (all *p* < 0.0001; Figure 8).

**Figure 9:**
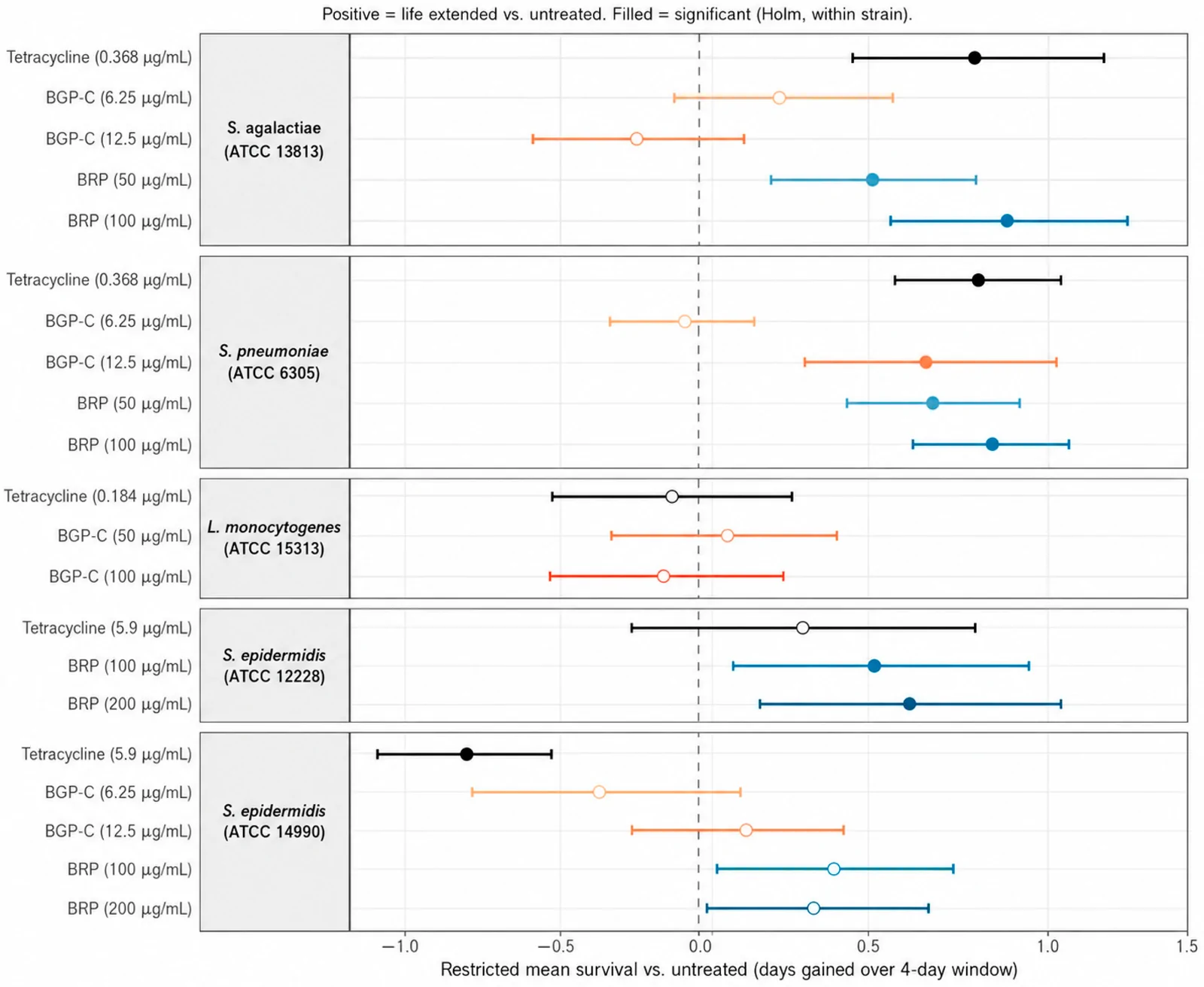
Effect of Brazilian red propolis (BRP), Brazilian propolis from the “Caatinga” (BGP-C), and tetracycline on the survival of *C. elegans* AU37 infected with different bacterial strains. Treatment effects are expressed as differences in restricted mean survival time (SΔRMST, days) relative to the untreated control over a 4-day observation period. Points represent ΔRMST estimates and horizontal bars indicate the 95% confidence intervals. Filled symbols indicate statistically significant differences after Holm-adjusted multiple comparisons, whereas open symbols indicate non-significant differences. The dashed vertical line represents no treatment effect (ΔRMST = 0). Black represents Tetracycline (positive control), orange indicates BGP-C and blue corresponds to BRP.

The therapeutic effects of BRP, BGP-C, and tetracycline were evaluated separately for each bacterial infection using ΔRMST as the primary efficacy endpoint (Figure 9 and Supplementary Material Table S2). Distinct treatment responses were observed among the evaluated bacterial infections.

BRP significantly prolonged survival in larvae infected with *S. agalactiae* (ATCC 13813) (100 μg, *p* < 0.0001; 50 μg, *p* = 0.0004), *S. pneumoniae* (ATCC 6305) (both concentrations, *p* < 0.0001), *S. epidermidis* (ATCC 12228) (200 μg, *p* = 0.0046; 100 μg, *p* = 0.016), and *S. epidermidis* (ATCC 14990) (200 μg, *p* = 0.036; 100 μg, *p* = 0.015).

In contrast, BGP-C provided a survival benefit only against *S. pneumoniae* (ATCC 6305) at 12.5 μg (*p* = 0.0001). No significant protection was observed against *S. agalactiae* (ATCC 13813), *S. epidermidis* (ATCC 12228), *S. epidermidis* (ATCC 14990), or *Listeria monocytogenes* (ATCC 15313) (*p* > 0.05).

Tetracycline also prolonged survival in larvae infected with *S. agalactiae* (ATCC 13813) and *S. pneumoniae* (ATCC 6305) (both *p* < 0.001) but had no effect against *L. monocytogenes* (ATCC 15313) (*p* = 0.57). Unexpectedly, tetracycline shortened survival in larvae infected with *S. epidermidis* (ATCC 14990) (*p* < 0.0001), whereas no significant effect was detected against *S. epidermidis* (ATCC 12228).

### 3.5. Cytotoxicity and Antiviral Activity Against ZIKV

#### 3.5.1. Cytotoxicity

To assess cytotoxicity in mammalian cells, Vero E6 cells were treated with each compound at concentrations of 50, 10, and 2 µg/mL. Cell viability was evaluated by the MTT assay after 72 h of incubation, with DMSO 0.1% used as the untreated control. Analysis of the effects on cell viability showed that compounds maintained viability above 80% at the concentrations of 10 and 2 µg/mL, indicating low cytotoxicity at the tested conditions (Supplementary Material, Figure S5). The highest non-cytotoxic concentration, 10 µg/mL, was therefore selected for evaluation of anti-ZIKV activity (Table 2).

Subsequently, cytotoxicity was also assessed in human villous trophoblast BeWo cells, an in vitro model for studying the human placenta and the maternal-fetal barrier. BeWo cells were treated with BGP-C and BRP at concentrations ranging from 100 to 0.2 µg/mL. Cell viability was evaluated by the MTT assay after 72 h of incubation, using 0.1% DMSO as the untreated control, allowing determination of the CC_50_ for each compound (Table 3). The cytotoxic profile could therefore be cell type-dependent. In Vero E6 cells, viability remained above 80% at 10 µg/mL, so that a CC_50_ could not be reached within this range, whereas BeWo cells were markedly more sensitive, with CC_50_ values of 5.7 µg/mL for BGP-C and 23.3 µg/mL for BRP. BGP-C was thus approximately four-fold more cytotoxic than BRP to trophoblast cells, and its CC_50_ lies below the highest concentration used in the Vero E6 screening (10 µg/mL), indicating that the non-cytotoxic range defined in Vero E6 cells cannot be extrapolated to the placental model.

#### 3.5.2. Antiviral Activity Against ZIKV

**Figure 10:**
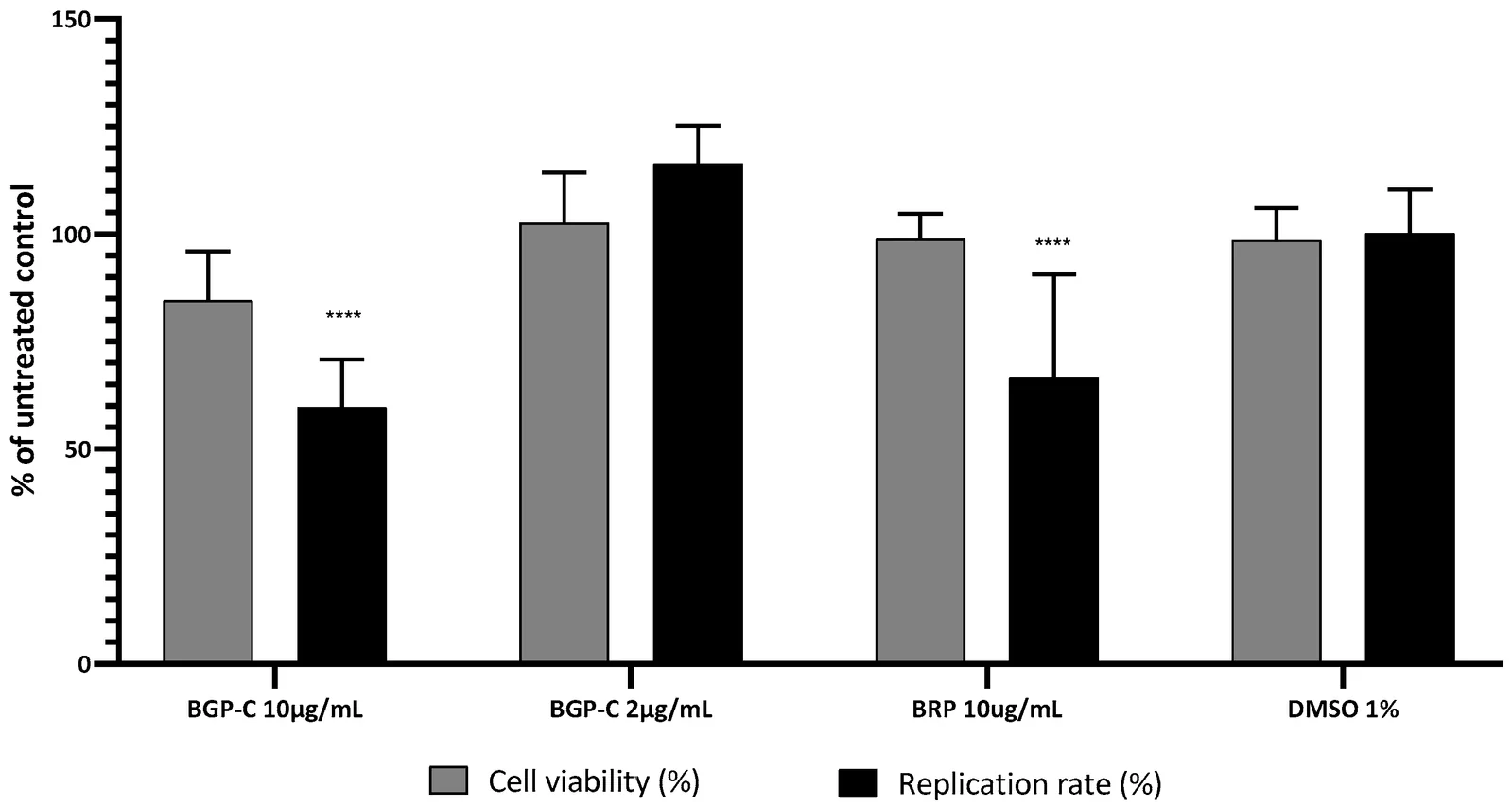
Effect of propolis compounds on Vero E6 cell viability and ZIKV infectivity. Vero E6 cells were infected with ZIKVPE243 at a multiplicity of infection (MOI) of 0.005 in the presence or absence of Brazilian green propolis from the “Caatinga” (BGP-C), and at an MOI of 0.01 in the presence or absence of Brazilian red propolis (BRP) at the highest non-cytotoxic concentration. After 72 h, cells were fixed and processed for the immunofluorescence assay. Infection foci (FFU/mL) were counted. Cell viability was measured by absorbance at 560 nm. DMSO was used as the untreated control. Mean values from two independent experiments, each performed in triplicate, including standard deviation, are shown. Values of *p* < 0.05 were considered statistically significant (****).

The anti-ZIKV activity of the natural compounds was initially investigated in Vero E6 cells infected with the ZIKVPE243 isolate. Cells were infected at an MOI of 0.005 in the presence or absence of BGP-C, and at an MOI of 0.01 in the presence or absence of BRP, using the highest non-cytotoxic concentration established. After 72 h, cells were fixed, washed with PBS, and incubated with blocking buffer for the immunofluorescence assay. FFU/mL were quantified by fluorescence microscopy using the EVOS cell imaging system (Thermo Fisher). The results showed that BGP-C and BRP, at a concentration of 10 μg/mL, inhibited ZIKV infection by 40.4% and 33.5%, respectively, compared with the untreated control (Table 2; Figure 10). These values correspond to a moderate reduction in viral infectivity obtained at a single non-cytotoxic concentration and should therefore be interpreted as preliminary evidence of anti-ZIKV activity of crude propolis extracts, rather than as an indication of a potent antiviral effect.

**Figure 11:**
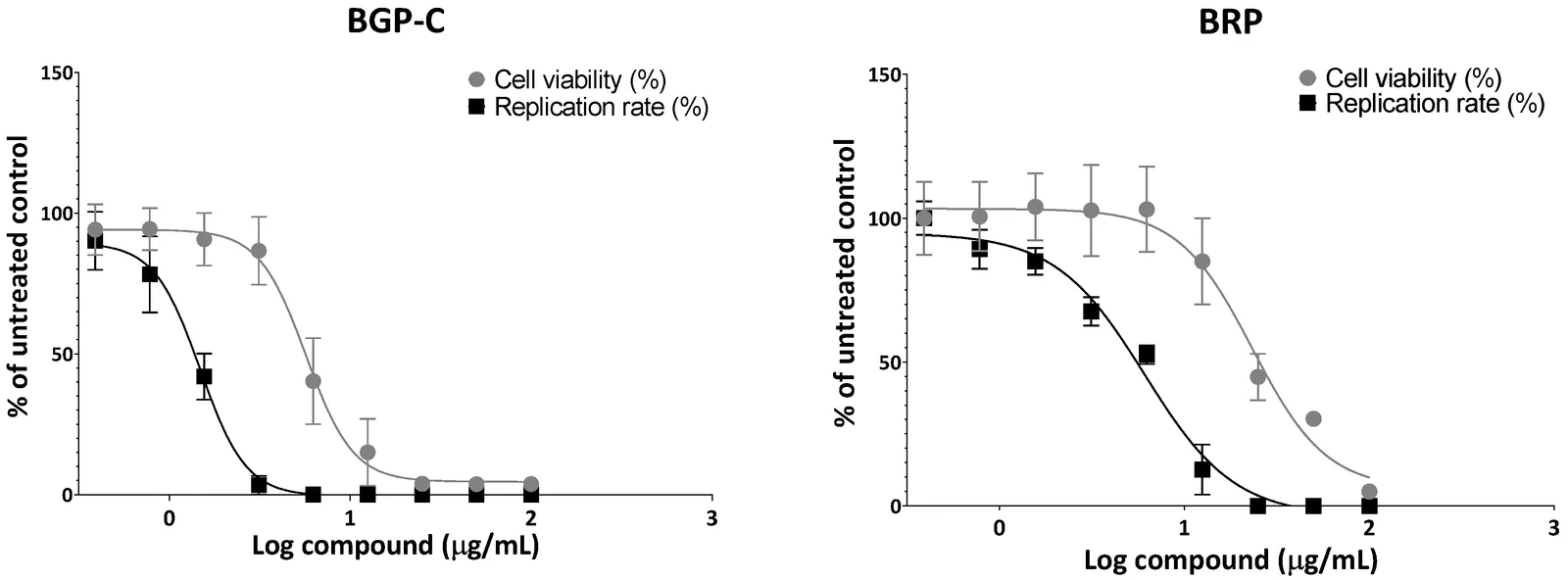
Dose–response curves of Brazilian red propolis (BRP) and Brazilian green propolis from “Caatinga” (BGP-C) on BeWo cell viability and ZIKV infectivity. BeWo cells were infected with ZIKVPE243 at an MOI of 0.01 in the presence or absence of compounds at concentrations ranging from 100 to 0.4 µg/mL. After 72 h, cells were fixed and processed for the immunofluorescence assay. Infection foci (FFU) were counted. Cell viability was measured by absorbance at 560 nm. DMSO (0.1%) was used as the untreated control. Mean values from two independent experiments, each performed in quadruplicate, including standard deviation, are shown.

Subsequently, anti-ZIKV activity was evaluated in BeWo cells infected with ZIKVPE243 at an MOI of 0.01, using the same concentration range of the compounds (100 to 0.1 μg/mL). After 72 h of infection, FFUs were quantified by immunofluorescence, allowing determination of the 50% effective concentrations (EC_50_) (Table 3; Figure 11).

#### 3.5.3. Selectivity Index in BeWo Cells

Based on cytotoxicity and antiviral assays of ZIKV performed in BeWo cells, selectivity indices (SI = CC_50_/EC_50_) were calculated. BGP-C showed a CC_50_ of 5.7 μg/mL, an EC_50_ of 1.5 μg/mL, and a selectivity index of 3.8. BRP presented a CC_50_ of 23.3 μg/mL, an EC_50_ of 6.1 μg/mL, and a selectivity index also of 3.8 (Table 3; Figure 11). An SI of 3.8 indicates measurable, reproducible selectivity for both extracts and is within the range commonly reported for crude natural product extracts prior to fractionation, in which antiviral and cytotoxic constituents typically coexist [54]. This value is nonetheless modest relative to the SI values often used to prioritize candidates for further development, indicating that the concentrations required to reduce ZIKV infectivity are relatively close to those affecting trophoblast viability, particularly for BGP-C, whose CC_50_ (5.7 µg/mL) and EC_50_ (1.5 µg/mL) differ by less than one order of magnitude. Rather than ruling out further investigation, this pattern is consistent with the expected profile of an unfractionated extract and supports bioguided fractionation as the next step to improve selectivity [21].

## 4. Discussion

Propolis is a resinous extract rich in flavonoids and phenolic acids and is widely recognized for its anti-inflammatory and antibacterial activities. However, its composition and biological potential vary according to botanical origin, geographical location, seasonality, and extraction methods, which explains the distinct chemical profiles observed among samples from different regions [23,24]. In this context, the investigation of propolis derived from still underexplored biomes becomes particularly relevant. In the present study, BRP extracts whose biological activity has been previously described in the literature but remains poorly explored against pathogens associated with neonatal infections, and BGP-C which has not been previously evaluated in this biological context, demonstrated activity against bacterial pathogens, and *Candida* spp. yeasts, and viruses associated with neonatal morbidity and mortality [1].

According to Rios and Recio (2005) [43], MIC values ≤ 100 µg/mL for crude extracts are considered promising in the evaluation of antibacterial activity. Although propolis is not a plant material per se, it is a natural product of indirect botanical origin and has been widely evaluated using similar criteria in antimicrobial studies. Following this criterion, the results obtained in the present study demonstrated relevant antibacterial activity of BRP and BGP-C extracts against bacteria frequently associated with neonatal infections, including *S. epidermidis* (ATCC 14990 and ATCC 12228), *S. agalactiae* (GBS, ATCC 13813), *S. pneumoniae* (ATCC 6305), and *L. monocytogenes* (ATCC 15313), pathogens of recognized clinical relevance in this context [2,13].

Previous studies have already demonstrated the antibacterial activity of Brazilian propolis extracts against Gram-positive bacteria. Salomao et al., (2008) [55], when evaluating ethanolic extracts of propolis collected from different Brazilian botanical sources, including *Baccharis dracunculifolia* and *Araucaria* spp., reported MIC values ranging from 0.2 to 0.8 µg/mL against *S. pneumoniae* (ATCC 49619) and from 1.6 to 52.4 µg/mL against *S. aureus* (ATCC 25923). Another study by de Souza Silva et al. (2021) [56], using Brazilian red propolis extract obtained from the Canavieiras region, Bahia state, and supplied by Apis Flora^®^ (Ribeirão Preto, São Paulo, Brazil), reported MIC values of 31.25 µg/mL for *S. epidermidis* (ATCC 14990) and 250 µg/mL for other *Staphylococcus* species. In the present study, although BRP from the same geographical region (Canavieiras, Bahia state, Brazil) was employed, the MIC values observed for the same strain were 100 µg/mL. This difference may reflect inherent variations related to seasonality, phytochemical composition, and the experimental conditions adopted, including methodological differences such as culture media and assay conditions, while still remaining comparable to those reported in the literature.

The activity observed against *L. monocytogenes* (ATCC 15313) deserves particular attention. Rendueles et al. (2023) [57], when evaluating 31 propolis extracts collected from different regions of Spain against multiple *Listeria* spp. strains, reported mean MIC values ranging from approximately 97 to 265 µg/mL, depending on the experimental conditions, with higher activity observed under acidic conditions. In the present study, BGP-C exhibited an MIC value of 50 µg/mL against *L. monocytogenes* (ATCC 15313), indicating notable antibacterial activity under standard experimental conditions. This difference may be attributed, at least in part, to specific features of BGP-C, whose botanical origin within the context of Brazil’s high plant biodiversity results in a unique phytochemical profile enriched in bioactive phenolic compounds, which may contribute to enhanced antimicrobial potency [30].

Beyond antibacterial effects, BRP and BGP-C extracts also demonstrated antibiofilm activity, representing one of the most relevant findings of this study, given that biofilm formation is a key virulence factor associated with bacterial persistence in the neonatal clinical setting. The antibiofilm activity of BRP has been previously reported against different microorganisms, including inhibition of oral biofilm formation at MBIC_50_ values ranging from 3.12 to 12.5 µg/mL [52], and against *Helicobacter pylori* (ATCC 43526) with an MBIC_50_ of 15.6 µg/mL [58]. Moreover, Martins et al. (2025) [59] demonstrated antibiofilm activity of BRP extracts against *Mycobacterium* spp., showing reductions in both biofilm biomass and metabolic activity at concentrations between 62.5 and 250 µg/mL, indicating that BRP retains activity even against structurally more complex microorganisms.

In studies conducted with propolis from different geographic origins, Wojtyczka et al. (2013) [60] demonstrated that ethanolic extracts of Polish propolis inhibited biofilm formation by different *S. epidermidis* strains at concentrations ranging from 390 to 1560 µg/mL. Similarly, Queiroga et al. (2023) [61] reported an average inhibition of 71% of staphylococcal biofilm formation using Brazilian brown propolis, achieved by exposing the isolates to half of the MBC. In contrast, in the present study, BRP showed MBIC_50_ values of 1.56 and 100 µg/mL against *S. epidermidis* strains ATCC 14990 and ATCC 12228, respectively, evidencing strain-dependent variability, as well as differences associated with the type of propolis evaluated and its geographic origin, since Brazilian red propolis presents a distinct phytochemical profile, associated with a higher richness of bioactive phenolic compounds when compared to brown propolis and samples from other regions. For BGP-C, although biomass reduction required higher concentrations, with an MBIC_50_ of 400 µg/mL for *S. epidermidis* (ATCC 14990), metabolic interference was observed at significantly lower concentrations, with an IC_50_ value of 4.77 µg/mL, indicating differential effects on biofilm and metabolic activity.

Fluorescent DAPI/PI staining showed visually increased PI-associated red fluorescence in biofilms treated with the propolis extracts compared with the untreated controls. Because PI preferentially enters cells with compromised membranes, this qualitative pattern is consistent with reduced cell viability and impaired bacterial membrane integrity [62]. SEM analyses complemented these findings by revealing extracellular matrix disorganization, microcolony disaggregation, and cellular surface alterations. This pattern is consistent with studies reporting, by fluorescence microscopy, an increase in PI-positive cells and, by SEM, structural changes in *Staphylococcus* biofilms treated with propolis extracts [50,63,64]. Similar microscopy-based approaches have been employed to investigate antimicrobial and antibiofilm effects. Wan et al. (2022) [65] demonstrated that a cationic photosensitizer–polypeptide conjugate enhanced binding to and penetration into *C. albicans* biofilms, with its antifungal and antibiofilm effects evaluated using complementary microscopy and quantitative biofilm analyses. More recently, Wan et al. (2026) demonstrated membrane disruption by tertiary alkylamine-functionalized polyaspartamides and quantitatively confirmed the leakage of intracellular ATP, DNA, and K^+^ [66]. In the present study, the increased PI-associated fluorescence and morphological alterations are therefore considered evidence consistent with compromised membrane integrity but do not establish membrane disruption as a definitive antibacterial mechanism. Future studies should include quantitative membrane-permeabilization assays, such as intracellular nucleic acid and protein leakage measurements, to confirm this proposed mechanism.

Detailed examination of SEM micrographs obtained at inhibitory concentrations revealed a pattern suggestive of propolis activity, characterized by microcolony disaggregation, reduced extracellular matrix, and morphological alterations on cell surfaces, highlighted by arrows and annotations in the figures for all analyzed species. In *S. epidermidis* (ATCC 14990 and ATCC 12228) and *S. agalactiae* (ATCC 13813), a visually apparent transition is observed from smooth, dense surfaces in control samples to less compact aggregates, with cells exhibiting rough contours and fissures, suggesting impairment of matrix integrity (Figure 5 and Figure 6). In the case of *S. pneumoniae* (ATCC 6305), fissure-like surface alterations and reduced cellular aggregation were observed, whereas in *L. monocytogenes* (ATCC 15313) a lower density of adhered cells predominates (Figure 6). These morphological findings are consistent with reports from studies using propolis or natural extracts that have demonstrated visible biofilm modifications, including morphological changes and partial matrix dispersion [50,64].

Despite some existing reports on the antimicrobial activity and chemical characterization of BGP-C, there remains a gap regarding the integrated evaluation of its antibacterial and antibiofilm activities. In this context, the present study provides an original contribution by combining quantitative metrics (MBIC_50_ and IC_50_) with morphological analyses by SEM, thereby expanding knowledge of the biological potential of this propolis against pathogens relevant to neonatal infections.

Beyond the antibacterial and antibiofilm activities previously demonstrated in vitro, the present study evaluated the therapeutic efficacy of Brazilian propolis extracts using the *C. elegans infection model*. This model represents an important intermediate platform between in vitro assays and mammalian studies because it enables the simultaneous evaluation of antimicrobial activity and host toxicity while preserving conserved innate immune pathways involved in host–pathogen interactions [67,68,69].

Before evaluating treatment efficacy, the virulence of clinically relevant neonatal pathogens was characterized in *C. elegans*. The bacterial species produced distinct survival profiles, demonstrating differences in pathogenic potential (Figure 8). This variability is consistent with the species-specific expression of virulence determinants, including adhesion factors, toxins, immune evasion mechanisms, and biofilm formation [70,71]. The inclusion of pathogens with different virulence profiles allowed the therapeutic efficacy of BRP to be evaluated under infections with different pathogenic potentials.

Among the evaluated pathogens, *S. epidermidis* deserves particular attention because biofilm formation is one of its principal virulence determinants. In the *C. elegans* model, biofilm-associated exopolysaccharides promote intestinal colonization and persistence, whereas disruption of biofilm-related genes reduces bacterial accumulation and significantly increases host survival [72]. In agreement with these findings, BRP significantly prolonged survival in both *S. epidermidis* strains (Figure 9). Considering its demonstrated antibiofilm activity, inhibition of biofilm formation may have contributed to the observed protection, although this mechanism was not directly investigated (Figure 2).

Although both BRP and BGP-C inhibited bacterial growth in vitro, their protective effects differed substantially in vivo. BRP consistently prolonged survival in infections caused by *S. epidermidis*, *S. pneumoniae*, and *S. agalactiae*, whereas BGP-C significantly improved survival only in the *S. pneumoniae* infection model (Figure 9). This contrast represents a key finding of the present study, indicating that the antibacterial activity observed in vitro did not translate into equivalent protective efficacy in vivo for the two propolis extracts. The broader protective activity of BRP may partly reflect its distinct phytochemical composition. HPLC-DAD analysis revealed the presence of isoflavonoids and prenylated phenolic compounds, including vestitol, neovestitol, medicarpin, oblongifolin B, and biochanin A, which have previously been associated with antibacterial and antibiofilm activities (Figure 1) [26,50].

Notably, all protective effects were observed at concentrations previously shown to be non-toxic to *C. elegans* (Figure 7), indicating that the increased survival was unlikely to result from nonspecific effects on host viability. These findings demonstrate that Brazilian red propolis exhibits consistent therapeutic activity against clinically relevant neonatal pathogens with distinct virulence profiles.

In addition to the antibiofilm and *C. elegans* infection assays, cytotoxicity was evaluated to establish a safe concentration range for the subsequent antiviral experiments. BGP-C and BRP exhibited a cell type–dependent cytotoxicity profile, with higher tolerance in Vero E6 cells, which maintained viability above 80% after 72 h of exposure to the tested concentrations. In contrast, human trophoblast BeWo cells were more sensitive, allowing determination of CC_50_ values and indicating a narrower therapeutic window, a behavior consistent with the higher susceptibility of placental cells to phenolic compounds reported in the literature [73,74]. Whereas Vero E6 cells tolerated concentrations up to 50 µg/mL with viability above 80%, BeWo cells showed CC_50_ values of 5.7 µg/mL for BGP-C and 23.3 µg/mL for BRP. For BGP-C in particular, the concentration used in the Vero E6 antiviral screening (10 µg/mL) already exceeds the CC_50_ determined in trophoblast cells, so that the therapeutic window in the placental model is narrow and the selectivity index remains modest. This possible cell type-dependent behavior may reflect differences in metabolic capacity, transporter expression and antioxidant defenses between the two lineages, and reinforces that non-cytotoxic ranges must be established independently for each cell model, particularly when the maternal-fetal interface is the intended target [75].

In screening assays using Vero E6 cells, both extracts moderately reduced the infectivity of the ZIKVPE243 isolate only at the highest non-cytotoxic concentration tested (10 µg/mL), with inhibition of approximately 40.4% for BGP-C and 33.5% for BRP. At lower concentrations, the antiviral effect was less pronounced, indicating a dose-dependent but limited activity in this cell line. This pattern of moderate effect is common for crude extracts, as chemical complexity may hinder discrimination between specific antiviral effects and nonspecific cytotoxic responses.

In a recent study, Mendonça et al. (2024) [76] evaluated the antiviral activity of an aqueous extract of stingless bee propolis in ZIKV-infected Vero cells. Antiviral activity was evaluated through the reduction in cytopathic effects and viral titers, as measured by the 50% Tissue Culture Infectious Dose (TCID_50_) assay, which quantifies the viral dose required to infect 50% of cell cultures. The authors reported an approximately 64-fold reduction in viral titers, corresponding to about 98% inhibition, when cells were pretreated with 10% (*v*/*v*) of the extract prior to infection, with no detectable cytotoxicity under the evaluated conditions.

Although these results indicate a more pronounced antiviral effect than that observed in the present study, direct quantitative comparison is limited by important methodological differences. Whereas the study by Mendonça et al. (2024) [76] employed concentrations expressed as volumetric proportions and evaluated antiviral activity through pretreatment and TCID_50_-based cytopathic effect assays, the present study used concentrations expressed in µg/mL and quantified antiviral activity by FFU analysis. These differences in extract preparation, dose units, and infection readout methods may substantially influence the magnitude of the observed antiviral effect. Nevertheless, both studies converge in indicating that propolis and geopropolis extracts contain constituents capable of interfering with the ZIKV replication cycle in cellular models.

The moderate magnitude of inhibition observed in Vero E6 cells and the intermediate selectivity index in BeWo cells (SI ≈ 3.8) are consistent with the use of crude extracts, in which compounds with antiviral and cytotoxic activities may coexist. Thus, the data indicate the presence of detectable anti-ZIKV activity, although with still limited selectivity, a pattern frequently reported for natural products evaluated prior to fractionation steps [21]. However, no reference antiviral compound was included as a positive control in the ZIKV assays and should be acknowledged as a limitation of the study.

## 5. Conclusions

This study demonstrates that BRP and BGP-C exhibit antibacterial activity against clinically relevant Gram-positive neonatal pathogens, inhibit biofilm formation, and show distinct therapeutic performances in the *C. elegans* infection model. BRP consistently improved larval survival across different bacterial infections, whereas BGP-C displayed a more limited in vivo protective effect, highlighting that antibacterial activity alone does not necessarily predict therapeutic efficacy. In addition, both extracts showed moderate and preliminary anti-ZIKV activity at non-cytotoxic concentrations, with a limited selectivity index in BeWo cells, so these results should be regarded as an initial screening outcome requiring confirmation with fractionated samples and reference antiviral controls. The phytochemical characterization and biological evaluation of BGP-C, a relatively underexplored Brazilian propolis, further expand current knowledge regarding its therapeutic potential. Collectively, these findings support BRP and BGP-C as promising sources of bioactive compounds for further investigation as potential adjuncts to conventional antimicrobial therapy, particularly for controlling bacterial biofilms. However, additional mechanistic, pharmacological, and clinical studies are required to establish their efficacy and safety in therapeutic applications.

## Figures and Tables

**Table 1:** Minimum Inhibitory Concentration, Minimum Bactericidal Concentration, and Minimum Fungicidal Concentration (MIC, MBC, and MFC) in µg/mL of Brazilian Green Propolis from the “Caatinga” (BGP-C) and Brazilian Red Propolis (BRP) extracts against microorganisms associated with neonatal infections included in the study.

Microorganisms	Samples	
	BGP-C	BRP
*Staphylococcus epidermidis* (ATCC 12228)	200/400	**100/>400**
*Staphylococcus epidermidis* (ATCC 14990)	**100/>400**	**100/>400**
*Neisseria gonorrhoeae* (ATCC 43069)	>400/>400	400/>400
*Escherichia coli* (ATCC BAA-198)	>400/>400	>400/>400
*Enterobacter cloacae* (clinical isolate IAL 124)	>400/>400	>400/>400
*Staphylococcus aureus* (ATCC 29213)	400/400	200/>400
*Streptococcus pneumoniae* (ATCC 6305)	**6.25/25**	**50/50**
*Listeria monocytogenes* (ATCC 15313)	**50/200**	200/>400
*Streptococcus agalactiae* (ATCC 13813)	**6.25/25**	**50/50**
*Streptococcus pyogenes* (ATCC 19615)	400/>400	400/>400
*Enterococcus faecalis* (ATCC 29212)	400/400	200/200
*Enterococcus faecalis* (ATCC 51299)	400/400	200/400
*Candida albicans* (ATCC 90028)	200/200	200/200

Control strains: *E. coli* (ATCC 25922)—MIC range: 0.25–1 µg/mL and *S. aureus* (ATCC 29213)—MIC range: 0.12–1 µg/mL. The MIC values obtained for gentamicin against these bacteria were 0.7375 µg/mL and 0.1844 µg/mL, respectively, both within the range recommended by the CLSI for these strains and antibiotic. Control strains for the antifungal assay: *C. krusei* (ATCC 6258)—MIC range: 0.5–2.0 µg/mL and *C. parapsilosis* (ATCC 22019)—MIC range: 0.25–1 µg/mL. The MIC value obtained for amphotericin B against these yeasts was 0.5 µg/mL for both, within the range recommended by the CLSI for these strains and antifungal agent. **Bold:** values indicate extracts selected for subsequent assays based on MIC values considered promising according to the criteria by Rios and Recio, 2005 [43].

**Table 2:** Effect of compounds from Brazilian green propolis from the “Caatinga” biome (BGP-C) and Brazilian red propolis (BRP) on Vero E6 cell viability and ZIKV infectivity (FFU/mL).

Compounds	Cell Viability (%)	ZIKV Infection (%)	ZIKV Replication Inhibition (%)
BGP-C 10 µg/mL	84.6	59.6	40.4
BRP 10 µg/mL	98.8	66.5	33.5

**Table 3:** The 50% cytotoxic concentration (CC_50_) and the 50% effective concentration (EC_50_) of propolis compounds on BeWo cell viability and ZIKV infectivity (FFU/mL). The selectivity index was calculated as SI = CC_50_/EC_50_.

Compounds	CC_50_ (µg/mL)	EC_50_ (µg/mL)	SI
Brazilian green própolis from the “Caatinga” (BGP-C)	5.7	1.5	3.8
Brazilian red propolis (BRP)	23.3	6.1	3.8

## Data Availability

All data supporting the findings of this study are included in the article and its Supplementary Materials. Additional data are available from the corresponding author upon reasonable request.

## Supplementary Materials

The following supporting information can be downloaded at: https://www.mdpi.com/article/10.3390/pathogens15090922/s1, The supplementary material includes the results of the positive controls for the antimicrobial and biofilm assays, figures showing the DAPI and IP fluorescence assays of biofilms treated with BRP and BGPC, statistical analyses of the survival curves in *C. elegans*, and the results of the cytotoxicity assay in Vero E6 cells. Table S1. Minimum Inhibitory Concentration and Minimum Bactericidal Concentration (MIC/MBC) in µg/mL of Tetracycline and Gentamicin against microorganisms associated with neonatal infections included in the study. Table S2. Treatment effects on *Caenorhabditis elegans* survival following bacterial infection, assessed by restricted mean survival time (RMST). Figure S1. Antibiofilm activity of Tetracycline monospecies biofilms formed by selected ATCC strains. Biofilm biomass and the metabolic activity of adhered cells were evaluated. (a) *Listeria monocytogenes* (ATCC 15313), (b) *Streptococcus agalactiae* (ATCC 13813), (c) *Staphylococcus epidermidis* (ATCC 14990), (d) *Staphylococcus epidermidis* (ATCC 12228) and *Streptococcus pneumoniae* (ATCC 6305). The minimum biofilm inhibitory concentration (MBIC_50_) was defined as the lowest extract concentration capable of reducing biofilm biomass by at least 50%. The concentration required to reduce the metabolic activity of adhered cells by 50% was defined as IC_50_. Figure S2. DAPI/PI fluorescence micrograph of biofilms of the strains in the study. Merged images allow visualization of viable cells (blue, DAPI) and non-viable cells with compromised membranes (red, propidium iodide, PI). Shown are untreated biofilm controls, biofilms treated with Brazilian green propolis from the “Caatinga” (BGP-C). (a) *S. epidermidis* (ATCC 14990); (b) *S. agalactiae* (ATCC 13813); (c) *S. pneumoniae* (ATCC 6305); (d) *L. monocytogenes* (ATCC 15313). Figure S3. DAPI/PI fluorescence micrographs of biofilms formed by the bacterial strains included in the study under control conditions and after treatment with Brazilian red propolis (BRP). Merged images allow visualization of viable cells (blue, DAPI) and non-viable cells with compromised membranes (red, propidium iodide, PI). (a) *S. epidermidis* (ATCC 14490); (b) *S. agalactiae* (ATCC 13813); (c) *S. pneumoniae* (ATCC 6305); (d) *S. epidermidis* (ATCC 12228). Figure S4. Kaplan–Meier survival curves of *C. elegans* AU37 infected with *S. epidermidis* (ATCC 12228), *S. epidermidis* (ATCC 14990), *S. pneumoniae* (ATCC 6305), *S. agalactiae* (ATCC 13813), and *L. monocytogenes* (ATCC 15313) in the absence of treatment. Survival differed significantly among bacterial strains, as determined by the log-rank test (χ^2^ = 67.0, df = 4, *p* < 0.0001). Figure S5. Effect of Brazilian green propolis from the “Caatinga” (BGP-C) and BRP Brazilian red propolis (BRP) on Vero E6 cells viability. Vero E6 cells were treated with compounds at 50, 10 and 2 µg/mL. After 72 h, absorbance (560 nm) was measured by MTT assay. DMSO 0.1% was used as untreated control. Mean values of three independent experiments, each measured in triplicate including the standard deviation are shown. *p* values < 0.05 were considered significant. **** *p* < 0.0001.

## Abbreviations

The following abbreviations are used in this manuscript:

>	Greater than
≈	Approximately
×	Multiplication
×10^n^	Scientific notation (base-10 exponent)
k×	Thousand-fold (microscopy magnification)
%	Percentage
(*v*/*v*)	Volume per volume
°C	Degree Celsius
µg	Microgram
µg/mL	Microgram per milliliter
µL	Microliter
µM	Micromolar
mg	Milligram
mg/mL	Milligram per milliliter
mL	Milliliter
nm	Nanometer
mm	Millimeter
h	Hour
rpm	Revolutions per minute
pH	Potential of hydrogen
ATCC	American Type Culture Collection
BHI	Brain Heart Infusion
BRP	Brazilian Red Propolis
BGP-C	Brazilian Green Propolis from the Caatinga
CC_50_	50% Cytotoxic Concentration
CFU	Colony-forming units
CFU/mL	Colony-forming units per milliliter
CLSI	Clinical and Laboratory Standards Institute
CO_2_	Carbon dioxide
DAPI	4′,6-diamidino-2-phenylindole
DNA	Deoxyribonucleic acid
DMSO	Dimethyl sulfoxide
OD	Optical density
EC_50_	50% Effective Concentration
FFU	Focus-forming units
FFU/mL	Focus-forming units per milliliter
GBS	Group B *Streptococcus* (*Streptococcus agalactiae*)
HPLC-DAD	High-Performance Liquid Chromatography with Diode Array Detection
IC_50_	50% Inhibitory Concentration
SEM	Scanning Electron Microscopy
MBC	Minimum Bactericidal Concentration
MBIC_50_	Minimum Biofilm Inhibitory Concentration resulting in a 50% reduction in biofilm biomass
MFC	Minimum Fungicidal Concentration
MIC	Minimum Inhibitory Concentration
MOI	Multiplicity of Infection
MTT	3-(4,5-dimethylthiazol-2-yl)-2,5-diphenyltetrazolium bromide
PBS	Phosphate-buffered saline
PI	Propidium iodide
PVC	Polyvinyl chloride
RMN	Nuclear Magnetic Resonance
RPMI	Roswell Park Memorial Institute medium
SDA	Sabouraud Dextrose Agar
SI	Selectivity Index
XTT	2,3-bis(2-methoxy-4-nitro-5-sulfophenyl)-5-[(phenylamino)carbonyl]-2H-tetrazolium hydroxide
ZIKV	Zika vírus

## References

[1] Yasin R., Jiang L., Das J.K., Bhutta Z.A. (2025). Interventions to Prevent and Manage Infections in Pregnancy. Neonatology.

[2] Raturi A., Chandran S. (2024). Neonatal Sepsis: Aetiology, Pathophysiology, Diagnostic Advances and Management Strategies. Clin. Med. Insights Pediatr..

[3] Tzialla C. (2025). Microbial Infections and Antimicrobial Use in Neonates and Infants. Trop. Med. Infect. Dis..

[4] Harrison M.L., Dickson B.F.R., Sharland M., Williams P.C.M. (2024). Beyond Early- and Late-Onset Neonatal Sepsis Definitions: What Are the Current Causes of Neonatal Sepsis Globally? A Systematic Review and Meta-Analysis of the Evidence. Pediatr. Infect. Dis. J..

[5] Rosa-Mangeret F., Benski A.C., Golaz A., Zala P.Z., Kyokan M., Wagner N., Muhe L.M., Pfister R.E. (2022). 2.5 Million Annual Deaths-Are Neonates in Low- and Middle-Income Countries Too Small to Be Seen? A Bottom-Up Overview on Neonatal Morbi-Mortality. Trop. Med. Infect. Dis..

[6] Rubio-Mora E., Bloise-Sánchez I., Quiles-Melero I., Cacho-Calvo J., Cendejas-Bueno E. (2025). Neonatal Sepsis: Epidemiology and Comparison between Preterm and Term Newborns. Enferm. Infecc. Y Microbiol. Clin. (Engl. Ed.).

[7] WHO SDG Target 3.2: End Preventable Deaths of Newborns and Children Under 5 Years of Age. https://www.who.int/news/item/01-02-2016-who-statement-on-the-first-meeting-of-the-international-health-regulations-(2005)-(ihr-2005)-emergency-committee-on-zika-virus-and-observed-increase-in-neurological-disorders-and-neonatal-malformations.

[8] Coggins S.A., Glaser K. (2022). Updates in Late-Onset Sepsis: Risk Assessment, Therapy, and Outcomes. NeoReviews.

[9] Falciglia G., Hageman J.R., Schreiber M., Alexander K. (2012). Antibiotic Therapy and Early Onset Sepsis. NeoReviews.

[10] Ferreira I., Menezes R.P., Lopes M.S.M., Araujo L.B., Ferreira D., Roder D. (2025). Epidemiology and Management of Infections in Critically Ill Neonates: Findings from a Cohort Study in a Brazilian Neonatal Intensive Care Unit. J. Med. Microbiol..

[11] Wen S.C.H., Ezure Y., Rolley L., Spurling G., Lau C.L., Riaz S., Paterson D.L., Irwin A.D. (2021). Gram-Negative Neonatal Sepsis in Low- and Lower-Middle-Income Countries and WHO Empirical Antibiotic Recommendations: A Systematic Review and Meta-Analysis. PLoS Med..

[12] Ferreira I.C.D.S., Machado I.C.D.B., Menezes R.D.P., Jesus T.A.D., Lopes M.S.M., Araújo L.B.D., Ferreira D.M.D.L.M., Röder D.V.D.D.B. (2025). Challenges and Trends in Gram-Negative Bacterial Infections in Critically Neonates: A Seven-and-a-Half-Year Observational Study. Am. J. Infect. Control.

[13] De Rose D.U., Ronchetti M.P., Martini L., Rechichi J., Iannetta M., Dotta A., Auriti C. (2024). Diagnosis and Management of Neonatal Bacterial Sepsis: Current Challenges and Future Perspectives. Trop. Med. Infect. Dis..

[14] Megli C.J., Carlin S.M., Giacobe E.J., Hillebrand G.H., Hooven T.A. (2025). Virulence and Pathogenicity of Group B *Streptococcus*: Virulence Factors and Their Roles in Perinatal Infection. Virulence.

[15] Russell N., Barday M., Okomo U., Dramowski A., Sharland M., Bekker A. (2024). Early-versus Late-Onset Sepsis in Neonates—Time to Shift the Paradigm?. Clin. Microbiol. Infect..

[16] Eletel L., Thomas T., Berry E.A., Kearns G.L. (2025). Emerging Treatments in Neonatal Fungal Infections: Progress and Prospects. Pediatr. Drugs.

[17] Khan F., Javaid A., Kim Y.-M. (2019). Functional Diversity of Quorum Sensing Receptors in Pathogenic Bacteria: Interspecies, Intraspecies and Interkingdom Level. Curr. Drug Targets.

[18] Zhang X., Zhang D., Zhou D., Zheng S., Li S., Hou Q., Li G., Han H. (2025). A Comprehensive Review of the Pathogenic Mechanisms of *Pseudomonas aeruginosa*: Synergistic Effects of Virulence Factors, Quorum Sensing, and Biofilm Formation. Front. Microbiol..

[19] Zhao A., Sun J., Liu Y. (2023). Understanding Bacterial Biofilms: From Definition to Treatment Strategies. Front. Cell. Infect. Microbiol..

[20] Al Beloushi M., Saleh H., Ahmed B., Konje J.C. (2024). Congenital and Perinatal Viral Infections: Consequences for the Mother and Fetus. Viruses.

[21] Pereira R.S., Santos F.C.P., Campana P.R.V., Costa V.V., De Pádua R.M., Souza D.G., Teixeira M.M., Braga F.C. (2023). Natural Products and Derivatives as Potential Zika Virus Inhibitors: A Comprehensive Review. Viruses.

[22] PAHO Zika: Symptoms, Prevention and Treatments. https://www.paho.org/en/topics/zika.

[23] Ayad A.S., Benchaabane S., Daas T., Smagghe G., Loucif-Ayad W. (2025). Propolis Stands out as a Multifaceted Natural Product: Meta-Analysis on Its Sources, Bioactivities, Applications, and Future Perspectives. Life.

[24] Lazo G.L., Lemes D.C., Pauletti P.M., Bastos J.K., Ambrosio S.R., Veneziani R.C.S., Pires R.H. (2025). Analysis of the Antimicrobial Activity of Propolis: A Narrative Review of in-vitro Studies. An. Acad. Bras. Ciênc..

[25] Ccana-Ccapatinta G.V., Mejia J.A.A., Tanimoto M.H., Groppo M., Carvalho J., Bastos J.K. (2020). *Dalbergia ecastaphyllum* (L.) Taub. and *Symphonia globulifera* L.f.: The Botanical Sources of Isoflavonoids and Benzophenones in Brazilian Red Propolis. Molecules.

[26] Felicio I.M., Cavalcanti A.M.T., Baranger K., de Oliveira Junior R.G., Poirot B., Picot L., de Andrade Cavalcante F. (2025). Brazilian Propolis: Chemical Composition, Regional Variability, and Bioactive Potential. Fitoterapia.

[27] Aldana-Mejia J.A., Ccana-Ccapatinta G.V., Squarisi I.S., Nascimento S., Tanimoto M.H., Ribeiro V.P., Arruda C., Nicolella H., Esperandim T., Ribeiro A.B. (2021). Nonclinical Toxicological Studies of Brazilian Red Propolis and Its Primary Botanical Source *Dalbergia ecastaphyllum*. Chem. Res. Toxicol..

[28] Santiago M.B., Leandro L.F., Rosa R.B., Silva M.V., Teixeira S.C., Servato J.P.S., Ambrósio S.R., Veneziani R.C.S., Aldana-Mejía J.A., Bastos J.K. (2022). Brazilian Red Propolis Presents Promising Anti-*H. Pylori* Activity in in vitro and in vivo Assays with the Ability to Modulate the Immune Response. Molecules.

[29] Ferreira J.M., Fernandes-Silva C.C., Salatino A., Negri G., Message D. (2017). New Propolis Type from North-East Brazil: Chemical Composition, Antioxidant Activity and Botanical Origin. J. Sci. Food Agric..

[30] Aldana-Mejia J.A., Ribeiro V.P., Katragunta K., Avula B., Tatapudi K.K., Bastos J.K., Khan I.A., Meepagala K., Ross S.A. (2024). Chemical Characterization and Antimicrobial Activity of Green Propolis from the Brazilian “Caatinga” Biome. Plants.

[31] Son N.T., Ribeiro V.P., Groppo M., Linh N.N., Bastos J.K. (2025). A Validated RP-HPLC–DAD Method for Analyzing Flavonoids in Caatinga Brazilian Green Propolis from *Mimosa tenuiflora* Produced by *Apis mellifera*. Rev. Bras. Farmacogn..

[32] da Silva C.C.F., Salatino A., da Motta L.B., Negri G., Salatino M.L.F. (2019). Chemical Characterization, Antioxidant and Anti-HIV Activities of a Brazilian Propolis from Ceará State. Rev. Bras. Farmacogn..

[33] Luyen N.D., Hao N.T., Bastos J.K., Ha N.X., Hung N.H., Gioi D.H., Linh N.N., Son N.T. (2024). Flavonoids from *Mimosa tenuiflora* Green Propolis: Chemotaxonomy, Antioxidant, Anti-Inflammation, Anti-Bacteria, Mosquito Larvicidal Activity, and In Silico Approach. ChemistrySelect.

[34] Clementino M.M., De Filippis I., Nascimento C.R., Branquinho R., Rocha C.L., Martins O.B. (2001). PCR Analyses of tRNA Intergenic Spacer, 16S-23S Internal Transcribed Spacer, and Randomly Amplified Polymorphic DNA Reveal Inter- and Intraspecific Relationships of *Enterobacter cloacae* Strains. J. Clin. Microbiol..

[35] (ATCC) American Type Culture Collection *Escherichia coli* (Migula) Castellani and Chalmers—ATCC BAA-198. https://www.atcc.org/products/baa-198.

[36] (ATCC) American Type Culture Collection *Enterococcus faecalis* (Andrewes and Horder) Schleifer and Kilpper-Balz—ATCC 51299. https://www.atcc.org/products/51299.

[37] Swenson J.M., Clark N.C., Sahm D.F., Ferraro M.J., Doern G., Hindler J., Jorgensen J.H., Pfaller M.A., Reller L.B., Weinstein M.P. (1995). Molecular Characterization and Multilaboratory Evaluation of *Enterococcus faecalis* ATCC 51299 for Quality Control of Screening Tests for Vancomycin and High-Level Aminoglycoside Resistance in Enterococci. J. Clin. Microbiol..

[38] Donald C.L., Brennan B., Cumberworth S.L., Rezelj V.V., Clark J.J., Cordeiro M.T., Freitas de Oliveira Franca R., Pena L.J., Wilkie G.S., Da Silva Filipe A. (2016). Full Genome Sequence and sfRNA Interferon Antagonist Activity of Zika Virus from Recife, Brazil. PLoS Neglected Trop. Dis..

[39] Cassani N.M., Santos I.A., Grosche V.R., Ferreira G.M., Guevara-Vega M., Rosa R.B., Pena L.J., Nicolau-Junior N., Cintra A.C.O., Mineo T.P. (2023). Roles of Bothrops Jararacussu Toxins I and II: Antiviral Findings against Zika Virus. Int. J. Biol. Macromol..

[40] Clinical and Laboratory Standards Institute CLSI (2010). Methods for Antimicrobial Dilution and Disk Susceptibility Testing of Infrequently Isolated or Fastidious Bacteria; Aproved Guideline—2nd ed..

[41] Sarker S.D., Nahar L., Kumarasamy Y. (2007). Microtitre Plate-Based Antibacterial Assay Incorporating Resazurin as an Indicator of Cell Growth, and Its Application in the in Vitro Antibacterial Screening of Phytochemicals. Methods.

[42] Clinical and Laboratory Standards Institute (2017). Reference Method for Broth Dilution Antifungal Susceptibility Testing of Yeasts: Approved Standard—Fourth Edition.

[43] Rios J.L., Recio M.C. (2005). Medicinal Plants and Antimicrobial Activity. J. Ethnopharmacol..

[44] Stepanovic S., Vukovic D., Hola V., Di Bonaventura G., Djukic S., Cirkovic I., Ruzicka F. (2007). Quantification of Biofilm in Microtiter Plates: Overview of Testing Conditions and Practical Recommendations for Assessment of Biofilm Production by Staphylococci. APMIS.

[45] O’Toole G.A. (2011). Microtiter Dish Biofilm Formation Assay. J. Vis. Exp..

[46] Wei G.X., Campagna A.N., Bobek L.A. (2006). Effect of MUC7 Peptides on the Growth of Bacteria and on *Streptococcus mutans* Biofilm. J. Antimicrob. Chemother..

[47] Pierce C.G., Uppuluri P., Tristan A.R., Wormley F.L., Mowat E., Ramage G., Lopez-Ribot J.L. (2008). A Simple and Reproducible 96-Well Plate-Based Method for the Formation of Fungal Biofilms and Its Application to Antifungal Susceptibility Testing. Nat. Protoc..

[48] Pierce C.G., Uppuluri P., Tummala S., Lopez-Ribot J.L. (2010). A 96 Well Microtiter Plate-Based Method for Monitoring Formation and Antifungal Susceptibility Testing of *Candida albicans* Biofilms. J. Vis. Exp..

[49] Williams S.C., Hong Y., Danavall D.C.A., Howard-Jones M.H., Gibson D., Frischer M.E., Verity P.G. (1998). Distinguishing between Living and Nonliving Bacteria: Evaluation of the Vital Stain Propidium Iodide and Its Combined Use with Molecular Probes in Aquatic Samples. J. Microbiol. Methods.

[50] Silva N.B.S., Calefi G.G., Teixeira S.C., Melo Fernandes T.A., Tanimoto M.H., Cassani N.M., Jardim A.C.G., Vasconcelos Ambrosio M.A.L., Veneziani R.C.S., Bastos J.K. (2024). Brazilian Red Propolis Reduces the Adhesion of Oral Biofilm Cells and the *Toxoplasma gondii* Intracellular Proliferation. Biomed. Pharmacother..

[51] Singulani J.D.L., Scorzoni L., Gomes P.C., Nazaré A.C., Polaquini C.R., Regasini L.O., Fusco-Almeida A.M., Mendes-Giannini M.J.S. (2017). Activity of Gallic Acid and Its Ester Derivatives in *Caenorhabditis elegans* and Zebrafish (*Danio Rerio*) Models. Future Med. Chem..

[52] Silva N.B.S., De Souza J.H., Santiago M.B., Da Silva Aguiar J.R., Martins D.O.S., Da Silva R.A., De Andrade Santos I., Aldana-Mejía J.A., Jardim A.C.G., Dos Santos Pedroso R. (2022). Potential in Vitro Anti-Periodontopathogenic, Anti-Chikungunya Activities and in Vivo Toxicity of Brazilian Red Propolis. Sci. Rep..

[53] Moy T.I., Conery A.L., Larkins-Ford J., Wu G., Mazitschek R., Casadei G., Lewis K., Carpenter A.E., Ausubel F.M. (2009). High-Throughput Screen for Novel Antimicrobials Using a Whole Animal Infection Model. ACS Chem. Biol..

[54] Artun F.T., Karagoz A., Ozcan G., Melikoglu G., Anil S., Kultur S., Sutlupinar N. (2016). In Vitro Anticancer and Cytotoxic Activities of Some Plant Extracts on HeLa and Vero Cell Lines. J. Buon.

[55] Salomao K., Pereira P.R., Campos L.C., Borba C.M., Cabello P.H., Marcucci M.C., de Castro S.L. (2008). Brazilian Propolis: Correlation between Chemical Composition and Antimicrobial Activity. Evid.-Based Complement. Altern. Med..

[56] de Souza Silva T., Silva J.M.B., Braun G.H., Mejia J.A.A., Ccapatinta G.V.C., Santos M.F.C., Tanimoto M.H., Bastos J.K., Parreira R.L.T., Orenha R.P. (2021). Green and Red Brazilian Propolis: Antimicrobial Potential and Anti-Virulence against ATCC and Clinically Isolated Multidrug-Resistant Bacteria. Chem. Biodivers..

[57] Rendueles E., Mauriz E., Sanz-Gomez J., Adanero-Jorge F., Garcia-Fernandez C. (2023). Antimicrobial Activity of Spanish Propolis against *Listeria monocytogenes* and Other Listeria Strains. Microorganisms.

[58] Santiago M.B., Tanimoto M.H., Ambrosio M.A.L.V., Veneziani R.C.S., Bastos J.K., Sabino-Silva R., Martins C.H.G. (2024). The Antibacterial Potential of Brazilian Red Propolis against the Formation and Eradication of Biofilm of *Helicobacter pylori*. Antibiotics.

[59] Martins S.B., Teixeira S.C., De Souza G., Alhatlani B.Y., Abdallah E.M., Ambrosio S.R., Silva T.S., Bastos J.K., Tanimoto M.H., Barbosa B.F. (2025). In Vitro Assessment of Brazilian Red Propolis against *Mycobacteria*: Antibacterial Potency, Synergy, Inhibition of Biofilm Formation, and Intramacrophage Effects. Front. Pharmacol..

[60] Wojtyczka R.D., Kępa M., Idzik D., Kubina R., Kabała-Dzik A., Dziedzic A., Wąsik T.J. (2013). In Vitro Antimicrobial Activity of Ethanolic Extract of Polish Propolis against Biofilm Forming *Staphylococcus epidermidis* Strains. Evid.-Based Complement. Altern. Med..

[61] Queiroga M.C., Laranjo M., Andrade N., Marques M., Costa A.R., Antunes C.M. (2023). Antimicrobial, Antibiofilm and Toxicological Assessment of Propolis. Antibiotics.

[62] Trinh K.T.L., Lee N.Y. (2022). Recent Methods for the Viability Assessment of Bacterial Pathogens: Advances, Challenges, and Future Perspectives. Pathogens.

[63] Grecka K., Xiong Z.R., Chen H., Pełka K., Worobo R.W., Szweda P. (2020). Effect of Ethanol Extracts of Propolis (EEPs) against Staphylococcal Biofilm—Microscopic Studies. Pathogens.

[64] Stahli A., Schroter H., Bullitta S., Serralutzu F., Dore A., Nietzsche S., Milia E., Sculean A., Eick S. (2021). In vitro Activity of Propolis on Oral Microorganisms and Biofilms. Antibiotics.

[65] Wan P., Guo W., Wang Y., Deng M., Xiao C., Chen X. (2022). Photosensitizer-Polypeptide Conjugate for Effective Elimination of *Candida albicans* Biofilm. Adv. Healthc. Mater..

[66] Wan P., Hua T., Zhao X., Deng M., Chen L., Xiao C., Chen X. (2026). Tertiary Alkylamine-Functionalized Polyaspartamides with Potent Antibacterial Activity. Bioact. Mater..

[67] Hunt P.R. (2017). The *C. elegans* Model in Toxicity Testing. J. Appl. Toxicol..

[68] Zarroug S.H.O., Bajaman J.S., Hamza F.N., Saleem R.A., Abdalla H.K. (2023). *Caenorhabditis elegans* as an in vivo Model for the Discovery and Development of Natural Plant-Based Antimicrobial Compounds. Pharmaceuticals.

[69] Irazoqui J.E., Urbach J.M., Ausubel F.M. (2010). Evolution of Host Innate Defence: Insights from *Caenorhabditis elegans* and Primitive Invertebrates. Nat. Rev. Immunol..

[70] Marsh E.K., May R.C. (2012). *Caenorhabditis elegans*, a Model Organism for Investigating Immunity. Appl. Environ. Microbiol..

[71] Darby C. (2005). Interactions with Microbial Pathogens. WormBook: The Online Review of C. elegans Biology.

[72] Begun J., Gaiani J.M., Rohde H., Mack D., Calderwood S.B., Ausubel F.M., Sifri C.D. (2007). Staphylococcal Biofilm Exopolysaccharide Protects against *Caenorhabditis elegans* Immune Defenses. PLoS Pathog..

[73] Dusza H.M., Van Boxel J., Van Duursen M.B.M., Forsberg M.M., Legler J., Vähäkangas K.H. (2023). Experimental Human Placental Models for Studying Uptake, Transport and Toxicity of Micro- and Nanoplastics. Sci. Total Environ..

[74] Myllynen P., Vähäkangas K. (2013). Placental Transfer and Metabolism: An Overview of the Experimental Models Utilizing Human Placental Tissue. Toxicol. In Vitro.

[75] Vicenti I., Boccuto A., Giannini A., Dragoni F., Saladini F., Zazzi M. (2018). Comparative Analysis of Different Cell Systems for Zika Virus (ZIKV) Propagation and Evaluation of Anti-ZIKV Compounds in Vitro. Virus Res..

[76] Mendonça R.Z., Nascimento R.M., Fernandes A.C.O., Silva P.I. (2024). Antiviral Action of Aqueous Extracts of Propolis from Scaptotrigona Aff. Postica (*Hymenoptera; Apidae*) against Zica, Chikungunya, and Mayaro Virus. Sci. Rep..

