# Supplementary material for: Brazilian Propolis in the Context of Pathogens Associated with Neonatal Infections: An Antibacterial and Antiviral Approach
Source: Pathogens. 2026 Sep 1;15(9):922. doi: 10.3390/pathogens15090922 (PMC13610924; doi:10.3390/pathogens15090922)
Supplement: Supplementary file 1 [file pathogens-15-00922-s001.zip › pathogens-4503406-supplementary.pdf]

pathogens

Article

Brazilian Propolis in the Context of Pathogens Associated with Neonatal Infections: An Antibacterial and Antiviral Approach

Anna Livia Oliveira Santos [1], Nagela Bernadelli Sousa Silva [1], Júlia Gomes Teixeira [1], Gabriel Guimarães Calefi [1], Júlia Amaral Vieira D'Almeida [2], Natasha Marques Cassani [2], Ana Carolina Gomes Jardim [2], Ralciane de Paula Menezes [3], Marcelo José Barbosa Silva [3], Matheus Hikaru Tanimoto [4], Victor Pena Ribeiro [4], Jairo Kenupp Bastos [4] and Carlos Henrique Gomes Martins [1,*]

Material Supplementary

Table S1. Minimum Inhibitory Concentration and Minimum Bactericidal Concentration (MIC/MBC) in μg/mL of Tetracycline and Gentamicin against microorganisms associated with neonatal infections included in the study.

MICRORGANISMS	Antibiotics	
	**Tetracycline**	**Gentamicin**
Staphylococcus epidermidis (ATCC 12228)	5.9/5.9	≤0.115/0.0461
Staphylococcus. epidermidis (ATCC 14990)	5.9/5.9	≤0.115/≤0.0115
Neisseria gonorrhoeae (ATCC 43069)	5.9/5.9	0.0461/01844
Escherichia coli (ATCC BAA-198)	0.7375/>5.9	0.0922/0.0922
Enterobacter cloacae (clinical isolated-IAL 124)	0.7375/0.7375	0.7375/>5.9
Staphylococcus aureus (ATCC 29213)	0.3688/>5.9	0.1844/5.9
Streptococcus pneumoniae (ATCC 6305)	0.461/0.3688	1.475/>5.9
Listeria monocytogenes (ATCC 15313)	0.1844/5.9	>5.9/>5.9
Streptococcus agalactiae (ATCC 13813)	0.461/0.3688	>5.9/>5.9
Streptococcus pyogenes (ATCC 19615)	0.3688/>5.9	>5.9/>5.9
Enterococcus faecalis (ATCC 29212)	>5.9/>5.9	>5.9/>5.9
Enterococcus. faecalis (ATCC 51299)	>5.9/>5.9	>5.9/>5.9
Candida albicans (ATCC 90028)	-	-

Technical control: *E. coli* (ATCC 25922) – MIC range: 0.25–1 μg/mL and *S. aureus* (ATCC 29213) – MIC range: 0.12–1 μg/mL. The MIC values obtained for gentamicin against these bacteria were 0.7375 μg/mL and 0.1844 μg/mL, respectively, both within the range recommended by the CLSI for these strains and antibiotic. Control strain for the antifungal assay: *C. krusei* (ATCC 6258) – MIC range: 0.5–2.0 μg/mL and *C. parapsilosis*.(ATCC 22019) -MIC range:0.25-1 μg/mL. The MIC value obtained for amphotericin B against these yeasts was 0.5 μg/mL for both, within the range recommended by the CLSI for these strains and antifungal agent.

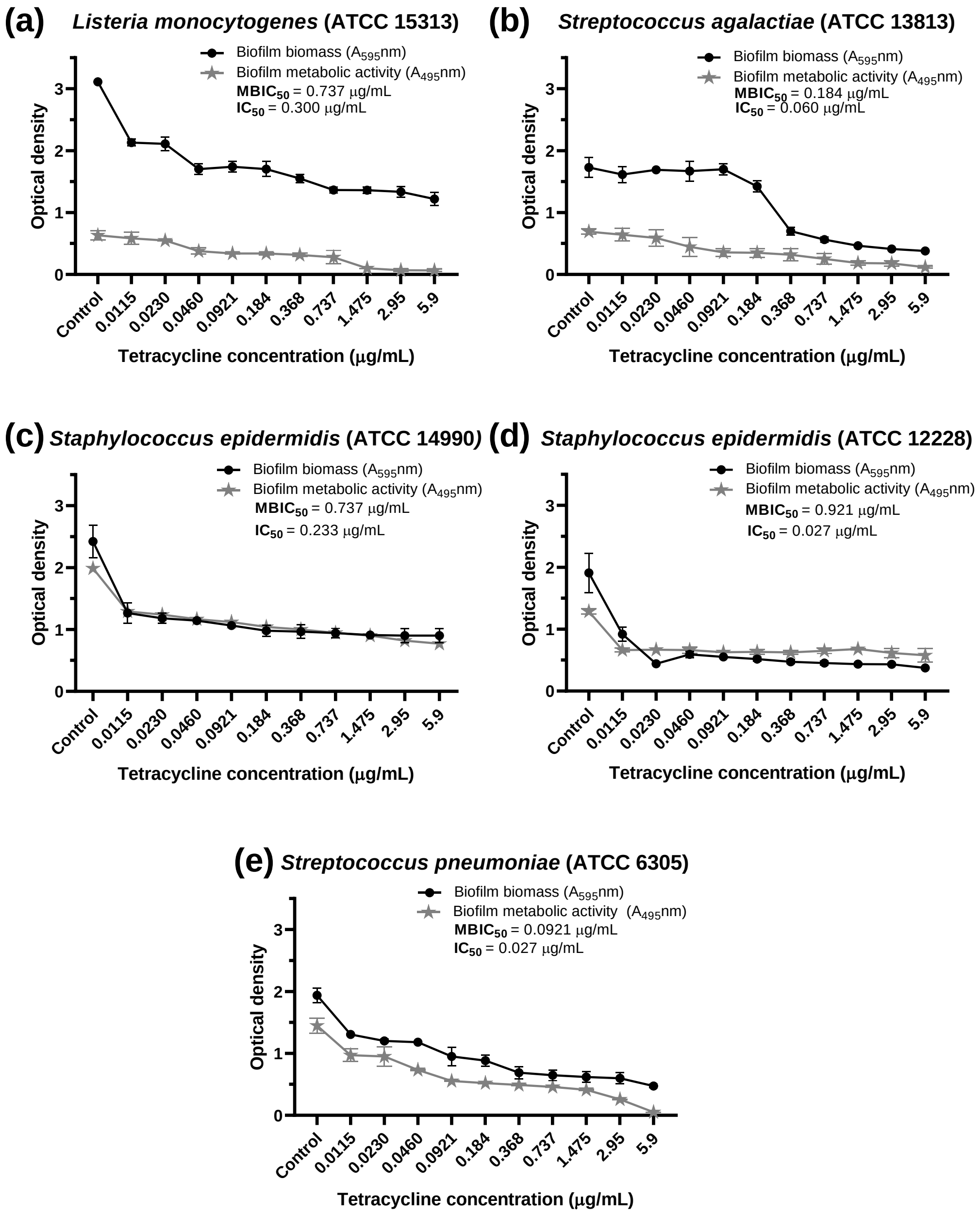

Figure S1. Antibiofilm activity of Tetracycline monospecies biofilms formed by selected ATCC strains. Biofilm biomass and the metabolic activity of adhered cells were evaluated. (a) *Listeria monocytogenes* (ATCC 15313), (b) *Streptococcus agalactiae* (ATCC 13813), (c*) Staphylococcus epidermidis* (ATCC 14990), (d) *Staphylococcus epidermidis* (ATCC 12228) and *Streptococcus pneumoniae* (ATCC 6305). The minimum biofilm inhibitory concentration (MBIC50) was defined as the lowest extract concentration capable of reducing biofilm biomass by at least 50%. The concentration required to reduce the metabolic activity of adhered cells by 50% was defined as IC50.

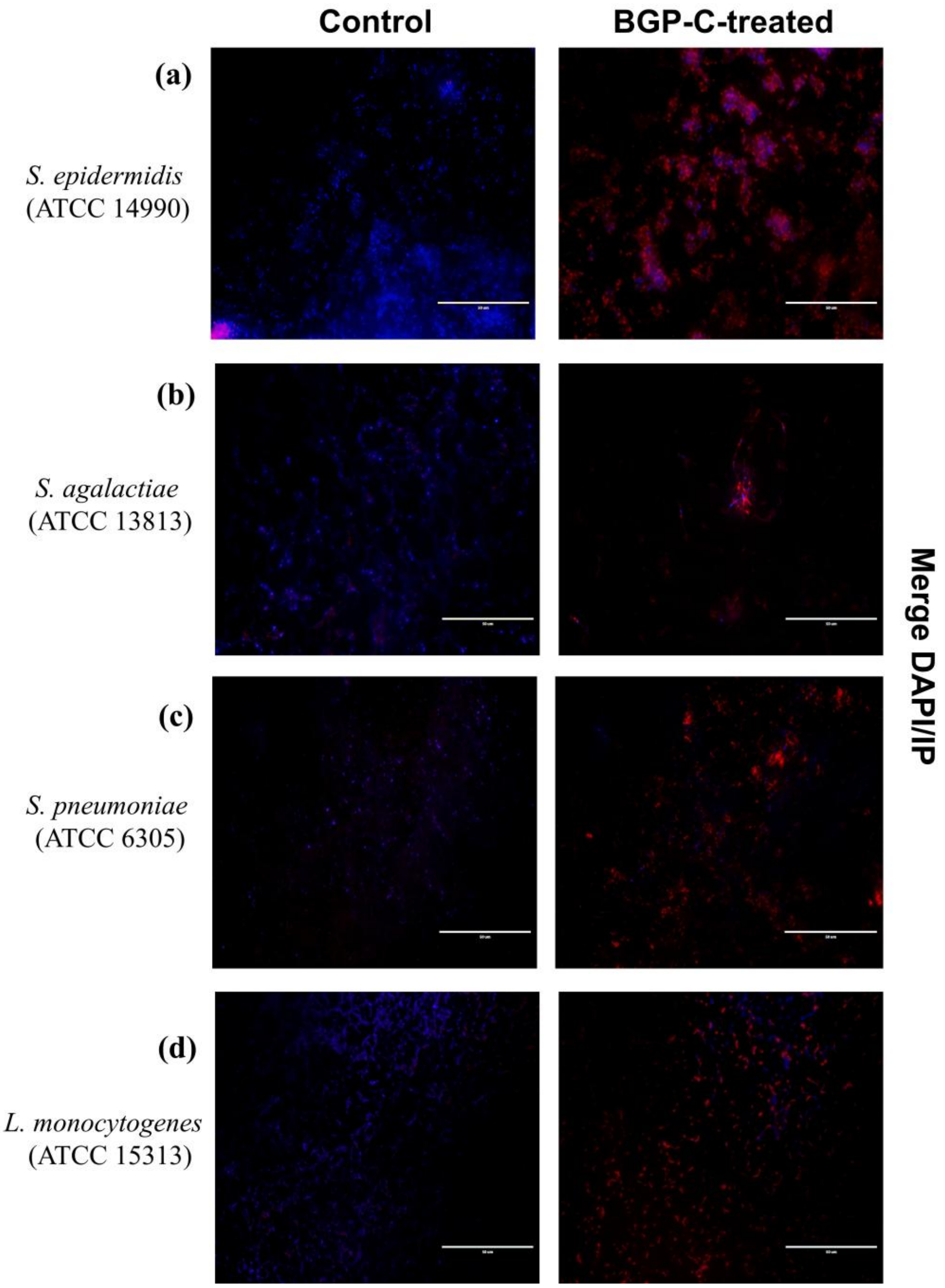

Figure S2. DAPI/PI fluorescence micrograph of biofilms of the strains in the study. Merged images allow visualization of viable cells (blue, DAPI) and non-viable cells with compromised membranes (red, propidium iodide, PI). Shown are untreated biofilm controls, biofilms treated with Brazilian green propolis from the "Caatinga" (BGP-C. a) *S. epidermidis* (ATCC 14990); b) *S. agalactiae* (ATCC 13813); (c) *S. pneumoniae* (ATCC 6305); d*)* *L. monocytogenes* (ATCC 15313).

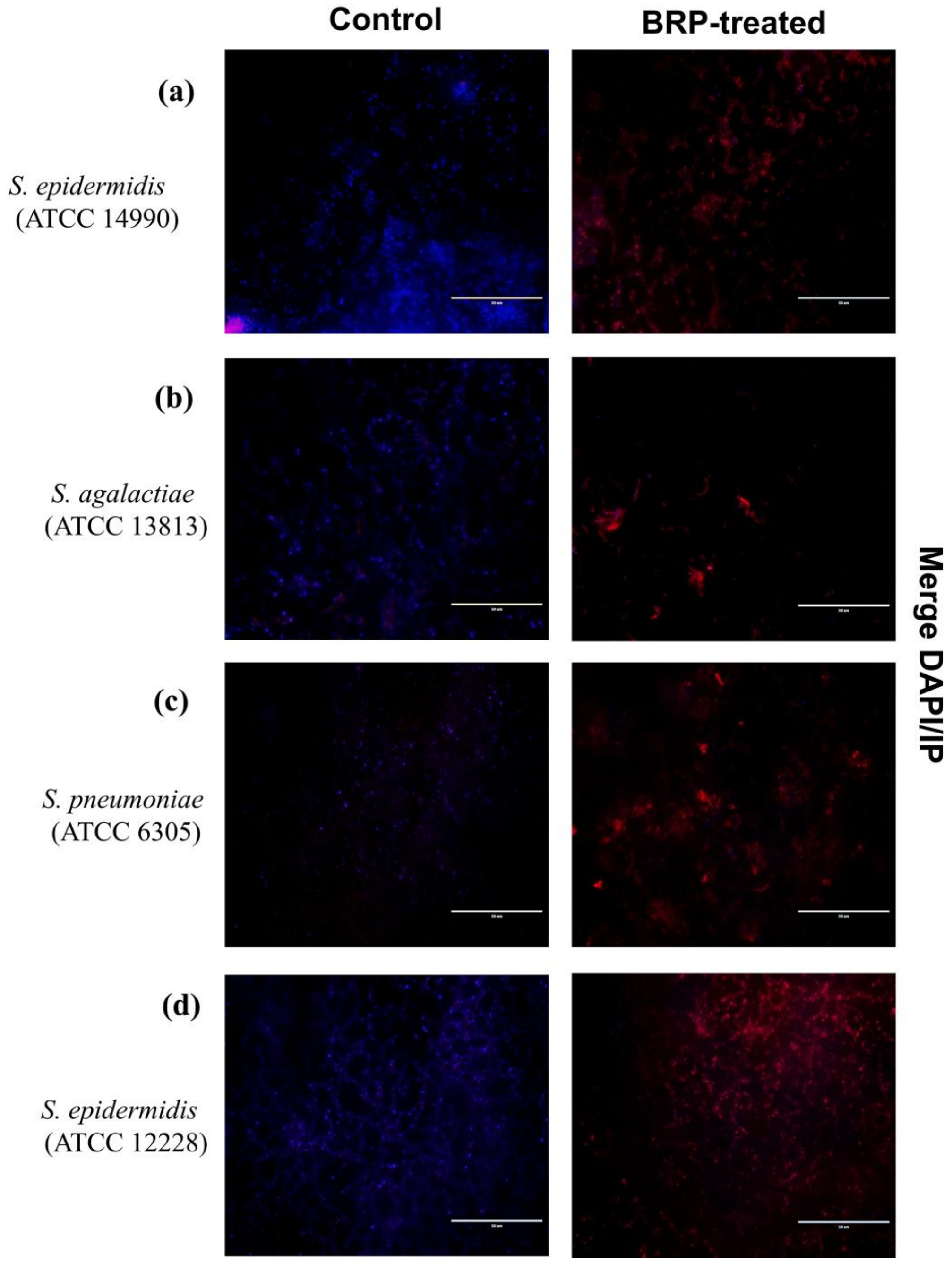

Figure S3. DAPI/PI fluorescence micrographs of biofilms formed by the bacterial strains included in the study under control conditions and after treatment with Brazilian red propolis (BRP). Merged images allow visualization of viable cells (blue, DAPI) and non-viable cells with compromised membranes (red, propidium iodide, PI). a) *S. epidermidis* (ATCC 14490); b) *S. agalactiae* (ATCC 13813); c) *S. pneumoniae* (ATCC 6305); d) *S. epidermidis* (ATCC 12228).

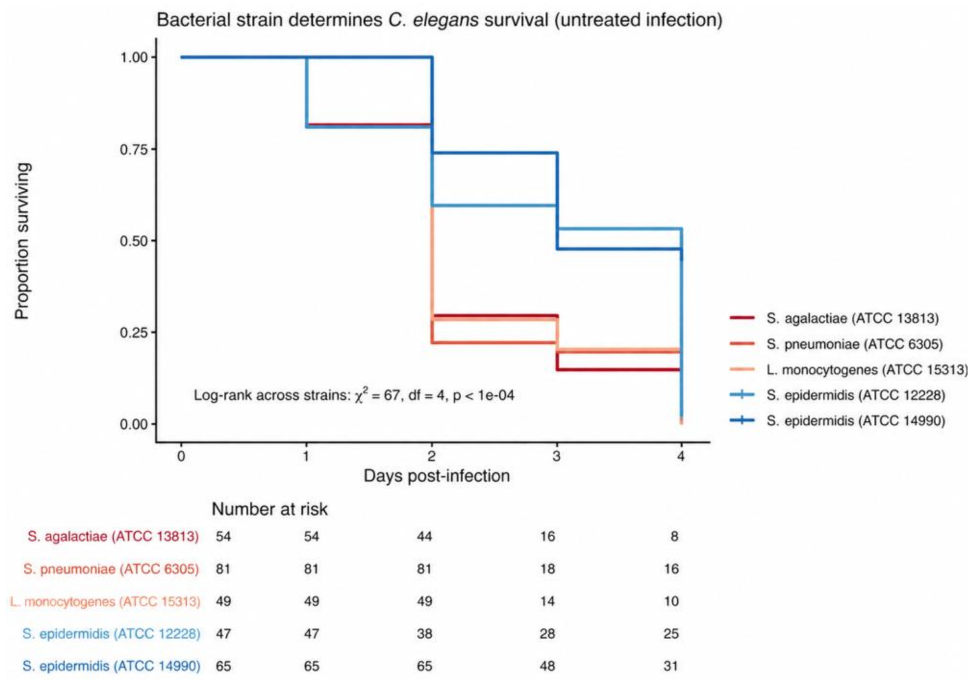

Figure S4. Kaplan–Meier survival curves of *C. elegans* AU37 infected with *S. epidermidis* (ATCC 12228), *S. epidermidis* (ATCC 14990), *S. pneumoniae* (ATCC 6305), *S. agalactiae* (ATCC 13813), and *L. monocytogenes* (ATCC 15313) in the absence of treatment. Survival differed significantly among bacterial strains, as determined by the log-rank test ($\chi^2 = 67.0$, df = 4, $p < 0.0001$).

Table S2. Treatment effects on *Caenorhabditis elegans* survival following bacterial infection, assessed by restricted mean survival time (RMST).

Bacterial strain	Treatment	Dose (µg/mL)	ΔRMST	95% CI	p value
***Streptococcus agalactiae* (ATCC 13813)**	BRP	100	+0.88	+0.55 to +1.20	<0.0001
	BRP	50	+0.55	+0.24 to +0.85	0.0004
	BGP-C	12.5	−0.12	−0.38 to +0.14	0.36
	BGP-C	6.25	+0.27	−0.03 to +0.56	0.075
	Tetracycline	0.461	+0.81	—	—
***Streptococcus pneumoniae* (ATCC 6305)**	BRP	100	+0.86	+0.65 to +1.08	<0.0001
	BRP	50	+0.69	+0.46 to +0.92	<0.0001
	BGP-C	12.5	+0.67	+0.33 to +1.01	0.0001
	BGP-C	6.25	−0.04	−0.25 to +0.18	0.75
	Tetracycline	0.461	+0.80	—	—
***Staphylococcus epidermidis* ATCC 12228**	BRP	200	+0.61	+0.19 to +1.03	0.0046
	BRP	100	+0.52	+0.10 to +0.94	0.016
	Tetracycline	5.9			
***Staphylococcus epidermidis* (ATCC 14990**	BRP	200	+0.33	+0.02 to +0.64	0.036
	BRP	100	+0.37	+0.07 to +0.66	0.015
	BGP-C	12.5	+0.10	−0.21 to +0.41	0.54
	BGP-C	6.25	−0.35	−0.79 to +0.10	0.13
	Tetracycline	5.9	−0.80	−1.04 to −0.55	<0.0001
***Listeria monocytogenes* (ATCC 15313)**	BGP-C	50	+0.06	−0.26 to +0.39	0.70
	BGP-C	100	−0.09	−0.41 to +0.22	0.56
	Tetracycline	0.1844	−0.11	—	0.50

ΔRMST represents the difference in restricted mean survival time between each treatment and the corresponding untreated infected control over the 4-day observation period (τ = 4 days). Positive ΔRMST values indicate prolonged survival, whereas negative values indicate reduced survival relative to the untreated infected control. Estimates are presented with 95% confidence intervals (95% CI), when available. p-values were adjusted for multiple comparisons using the Holm method within each bacterial strain. Treatment concentrations are expressed in µg/mL. BGP-C, Brazilian green propolis from the "Caatinga"; BRP, Brazilian red propolis; RMST, restricted mean survival time; CI, confidence interval.

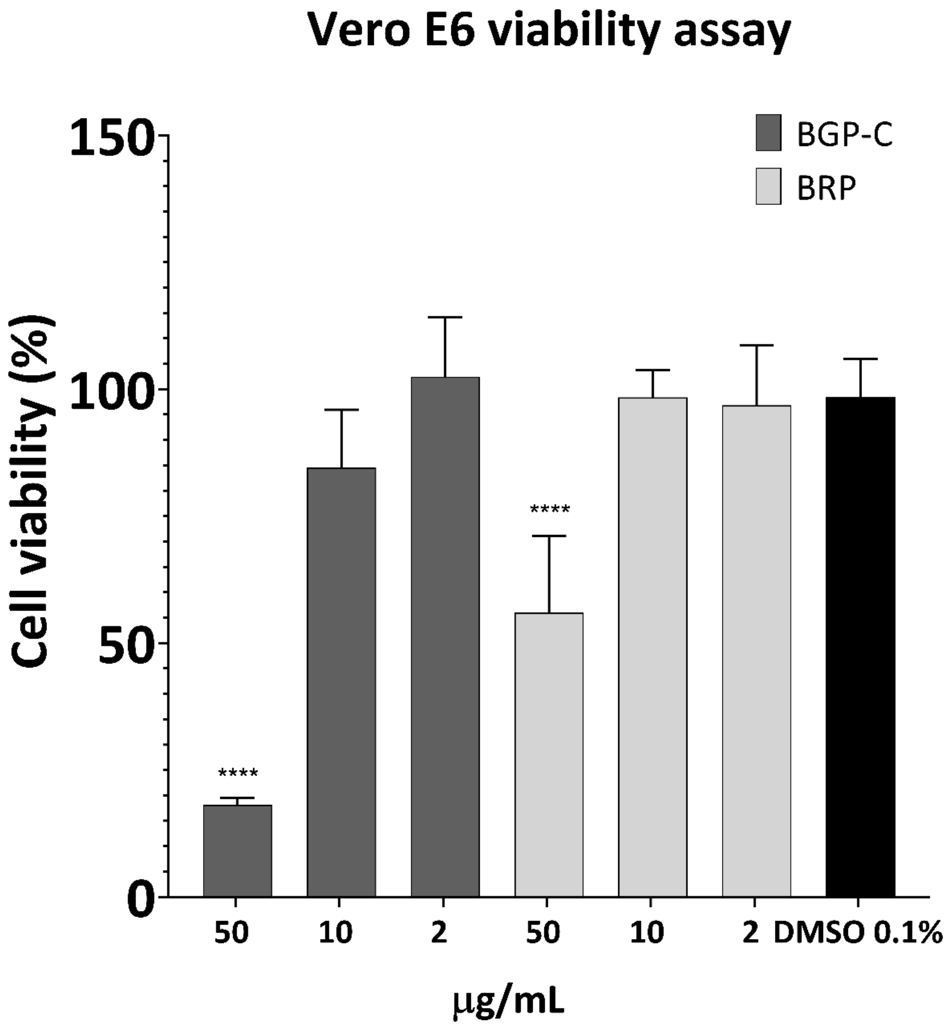

Figure S5. Effect of Brazilian green propolis from the "Caatinga" (BGP-C) and BRP Brazilian red propolis (BRP) on Vero E6 cells viability. Vero E6 cells were treated with compounds at 50, 10 and 2 µg/mL. After 72 h, absorbance (560 nm) was measured by MTT assay. DMSO 0.1% was used as untreated control. Mean values of three independent experiments, each measured in triplicate including the standard deviation are shown. P values < 0.05 were considered significant. **** $p < 0.0001$.
